# A New Meroterpene, A New Benzofuran Derivative and Other Constituents from Cultures of the Marine Sponge-Associated Fungus *Acremonium persicinum* KUFA 1007 and Their Anticholinesterase Activities

**DOI:** 10.3390/md17060379

**Published:** 2019-06-25

**Authors:** Ana J. S. Alves, José A. Pereira, Tida Dethoup, Sara Cravo, Sharad Mistry, Artur M. S. Silva, Madalena M. M. Pinto, Anake Kijjoa

**Affiliations:** 1 Laboratório de Química Orgânica, Departamento de Ciências Químicas, Faculdade de Farmácia, Universidade do Porto, Rua de Jorge Viterbo Ferreira, 228, 4050-313 Porto, Portugal; anajoao93@hotmail.com (A.J.S.A.); scravo@ff.up.pt (S.C.); 2 *ICBAS*-Instituto de Ciências Biomédicas Abel Salazar, Rua de Jorge Viterbo Ferreira, 228, 4050-313 Porto, Portugal; pereira@icbas.up.pt; 3 Interdisciplinary Centre of Marine and Environmental Research (CIIMAR), Terminal de Cruzeiros do Porto de Lexões, Av. General Norton de Matos s/n, 4450-208 Matosinhos, Portugal; 4 Department of Plant Pathology, Faculty of Agriculture, Kasetsart University, Bangkok 10240, Thailand; tdethoup@yahoo.com; 5 Department of Chemistry, University of Leicester, University Road, Leicester LE 7 RH, UK; scm11@leicester.ac.uk; 6 Departamento de Química & QOPNA, Universidade de Aveiro, 3810-193 Aveiro, Portugal; artur.silva@ua.pt

**Keywords:** *Acremonium persicinum*, Hypocreaceae, marine sponge-associated fungus, meroterpene, acremine S, acremine T, benzofuran, anticholinesterase activities

## Abstract

Previously unreported meroterpene, acremine S (**1**), and benzopyran derivative, acremine T (**2**), were isolated, together with lumichrome (**3**), ergosterol (**4**) and ergosterol 5,8-endoperoxide, from cultures of the marine sponge-associated fungus *Acremonium persicinum* KUF1007. The structure of the previously unreported compounds was established based on an extensive analysis of 1D and 2D NMR spectra as well as HRMS data. The absolute configurations of the stereogenic centers of **1** were established, unambiguously, based on NOESY correlations and comparison of calculated and experimental electronic circular dichroism (ECD) spectra. Compounds **1–3** were tested for their *in vitro* acetylcholinesterase and butyrylcholinesterase inhibitory activities.

## 1. Introduction 

The genus *Acremonium* (Hypocreaceae) includes approximately 100 species and are known as saprobic on dead plants or soil dwellers [1]. Many species of this genus have been identified as producers of structurally and biologically diverse secondary metabolites [2] including hydroquinone inhibitors of neutral sphingomyelinase (N-SMase) [3], anti-phytopathogenic meroterpenoids [4,5,6], as well as the enzyme cellulase [7]. Marine-derived species of *Acremonium* have also been investigated for their secondary metabolites as exemplified by a sponge-derived *Acremonium* sp. which produced anti-inflammatory sesquiterpenoids [8], and a mangrove-derived *Acremonium* sp. which produced phthalide and isocoumarin derivatives [9]. Concerning *Acremonium persicinum*, both marine-derived and soil-derived strains have shown to be interesting sources of structurally variable secondary metabolites. Suciati et al. [10] reported the isolation of several previously described acremines and spiroacremines as well as new acremines and chloroacremines from *A. persicinum*, isolated from the marine sponge *Anomoianthella rubra*. Recently, Nakamura et al. [11] reported a novel antifungal hexapeptide, ASP2397, from *A. persicinum* MF-347833, isolated from leaf litter, collected at Endau Rompin National Park in Malaysia, while Wang et al. [12] described the isolation of four peptaibiotics, acremotins A–D, from the culture of the soil-derived fungus *A. persicinum* SC0105.

Recently, much attention has been paid to marine-derived compounds with potential for treatment of neurological disorders such as Alzheimer’s diseases (AD) [13]. Acetylcholinesterase (AChE) and butyrylcholine esterase (BuChE), which break down acetylcholine and butyrylcholine, are considered as promising targets in the management of AD. Surprisingly, only a few novel AChE inhibitors were reported from marine-derived fungi. Examples of these are the oxylipin, (8E, 12*Z*)-10,11-dihydroxyoctadeca-8,12-dienoic acid, and a steroid, 3β,4α-dihydroxy-26-methoxyergosta-7,24 (28)-diene-6-one, obtained from the cultures of an endophytic fungus, *Aspergillus flavus*, which possessed low activity to inhibit AChE [14]. On the other hand, Wu et al. [15] have found that the oxaphenalenone dimers, talaromycesones A and B, as well as the isopentenyl xanthenone, talaroxanthenone, displayed potent acetylcholinesterase inhibitory activities. Liu et al. [16] have found that the ethyl acetate extract of the cultures of *Aspergillus ochraceus* SH0701, isolated from the sea sediment in China, potently inhibited acetylcholinesterase; however, they did not isolate the compounds from this active extract.

Inspired by these few and recent findings, thus, in our ongoing search for new bioactive compounds from marine-derived fungi, from the Gulf of Thailand, we investigated secondary metabolites from the cultures of *A. persicinum* KUFA1007, which was isolated from the marine sponge *Mycale* sp., collected from the coral reef at Samaesarn Island, Chonburi Province, in the Gulf of Thailand. The isolated compounds were assayed for their capacity to inhibit the enzymes AChE and BuChE.

**Figure 1:**
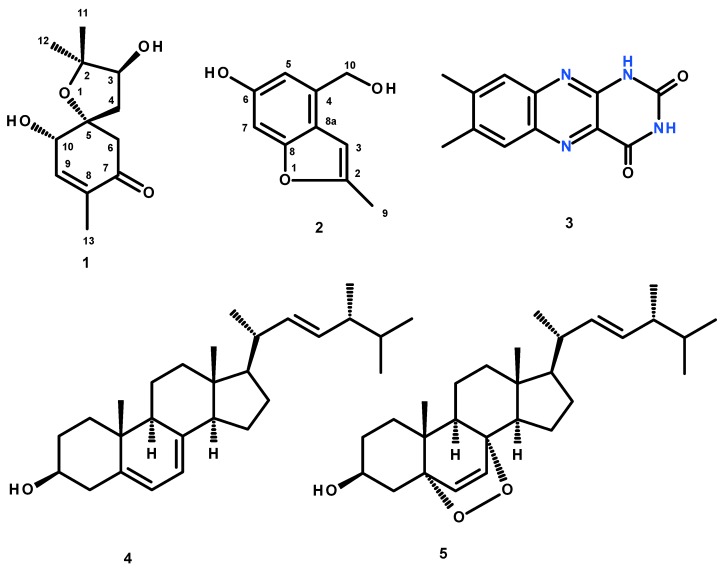
Structures of secondary metabolites isolated from the cultures of the marine sponge-associated fungus *Acremonium persicinum* KUFA1007.

Fractionation of the ethyl acetate crude extract of the cultures of *A. persicinum* KUFA007 by column chromatography, followed by purification by crystallization, preparative TLC and Sephadex LH-20 column, furnished previously undescribed spiroacremine (**1**) and benzofuran derivative (**2**), in addition to the previously reported lumichrome (**3**), ergosterol (**4**) and ergosterol 5,8-endoperoxide (**5**) (Figure 1). The structures of the previously undescribed compounds were established based on extensive analysis of their 1D and 2D NMR as well as HRMS data while the identity of the known compounds were elucidated by comparison of their ^1^H and ^13^C NMR data with those of the authentic samples as well as from the literature. Compounds **1**–**3** were tested for their anticholinesterase activity against AChE and BuChE by the modified Ellman’s method.

## 2. Results and Discussion 

The structures of lumichrome (**3**) [17,18,19], ergosterol (**4**) [20], and ergosterol 5,8-endoperoxide (**5**) [21] (Figure 1) were elucidated by analysis of their 1D and 2D NMR spectra as well as HRMS data, and also by comparison of their spectral data (Figures S23–S33, Table S1) with those reported in the literature. 

**Figure 2:**
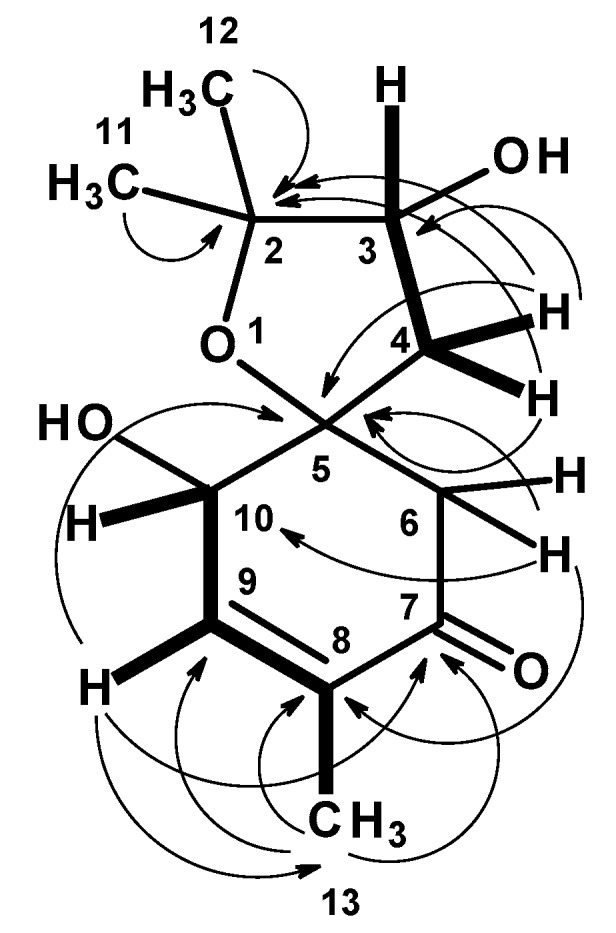
Key COSY (bold line) and HMBC (arrow) correlations in **1**.

Compound **1** was isolated as a yellowish viscous oil, and its molecular formula C_12_H_18_O_4_ was established based on the (+)-HRESIMS *m/z* 227.1293 [M + H]^+^ (calculated 227.1283) (Figure S10), indicating four degrees of unsaturation. The ^13^C NMR spectrum, in combination with DEPT and HSQC spectra (Table 1, Figures S2–S4 and S6), exhibited twelve carbon signals, which can be categorized as one conjugated ketone carbonyl (δ_C_ 197.8), one quaternary sp^2^ (δ_C_ 135.6), one methine sp^2^ (δ_C_ 147.2), two oxyquaternary sp^3^ (δ_C_ 87.1 and 85.8), two oxymethine sp^3^ (δ_C_ 77.3 and 73.6), two methylene sp^3^ (δ_C_ 53.0 and 39.4) and three methyl (δ_C_ 27.8, 22.6 and 15.0) carbons. The ^1^H NMR spectrum (Table 1, Figure S1), in combination with the HSQC spectrum (Table 1, Figure S6), revealed the presence of a triplet of an olefinic proton at δ_H_ 6.58 (*J* = 1.6 Hz; δ_C_ 147.2), a triplet of an oxymethine proton at δ_H_ 4.56 (*J* = 2.1 Hz; δ_C_ 73.6), a double doublet of another oxymethine proton at δ_H_ 3.90 (*J* = 4.3, 1.9 Hz; δ_C_ 77.3), two doublets of geminally coupled methylene protons at δ_H_ 2.67 (*J* = 15.5 Hz; δ_C_ 53.0) and δ_H_ 2.77 (*J* = 15.5 Hz; δ_C_ 53.0), a multiplet of two methylene protons at δ_H_ 2.20 (δ_C_ 39.4), a double doublet of methyl protons at δ_H_ 1.78 (*J* = 2.1, 1.6 Hz, δ_C_ 39.4), and two methyl singlets at δ_H_ 1.31 (δ_C_ 22.6) and δ_H_ 1.20 (δ_C_ 27.8), respectively. The COSY spectrum (Table 1, Figure S5, Figure 2) displayed correlations from the olefinic proton at δ_H_ 6.58 ( *J* = 1.6 Hz, H-9) to the triplet at δ_H_ 4.56 (*J* = 2.1 Hz; H-10) and the methyl double doublet at δ_H_ 1.78 (*J* = 2.0, 1.6 Hz, Me-13), while the HMBC spectrum (Table 1, Figure S7, Figure 2) showed correlations from H_3_-13 to the carbonyl carbon at δ_C_ 197.8 (C-7) and the olefinic carbons at δ_C_ 147.2 (C-9) and 135.6 (C-8), H-9 to C-7, the oxyquaternary sp^3^ carbon at δ_C_ 87.1 (C-5) and the methyl carbon at δ_C_ 15.0 (Me-13), the doublets at δ_H_ 2.67 and 2.77 (H_2_-6) to the carbon at δ_C_ 73.6 (C-10), C-8, C-7 and C-5. Taking together the ^1^H and ^13^C chemical shift values and the COSY and HMBC correlations, the partial structure of the molecule was established as 4-hydroxy-2-methylcyclohex-2-en-1-one. Another portion of the molecule was evidenced to be 2,2-dimethyloxolan-3-ol by the COSY correlation (Table 1, Figure 2 and Figure S5) from the multiplet at δ_H_ 2.20 (H_2_-4) to the double doublet at δ_H_ 3.90 (H-3), as well as the HMBC correlations (Table 1, Figure 2 and Figure S7) from H_2_-4 to the carbon signals at δ_C_ 77.3 (C-3), 85.8 (C-2), C-5, the methyl singlet at δ_H_ 1.20 (H_3_-11) to C-2, C-3 and the methyl carbon at δ_C_ 22.6 (Me-12), the methyl singlet at δ_H_ 1.31 (H_3_-12) to C-2, C-3 and the methyl carbon at δ_C_ 27.8 (Me-11). That the 2, 2-dimethyloxolan-3-ol was *spiro*-fused with the 4-hydroxy-2-methylcyclohex-2-en-1-one moiety at C-5 was corroborated by the HMBC correlation (Table 1, Figure S7, Figure 2) from H_2_-4 to C-5, C-6 and C-10 as well as from H_2_-6 to C-4 and C-5. Taken together the two partial structures and the HMBC correlations, the planar structure of **1** was established as 3,10-dihydroxy-2,2,8-trimethyl-1-oxospiro[4.5]dec-8-en-7-one. 

**Figure 3:**
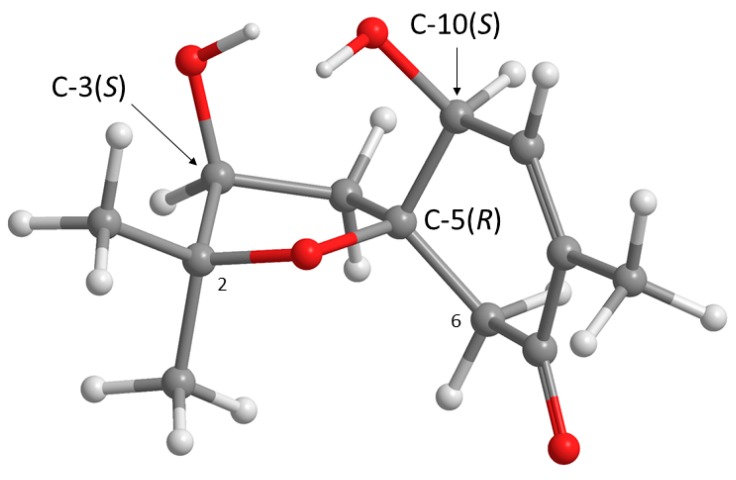
Most abundant conformation of **1** (APFD/6-311+G(2d,p) lowest energy conformer) in its (3*S*, 5*R*, 10*S*) configuration.

**Figure 4:**
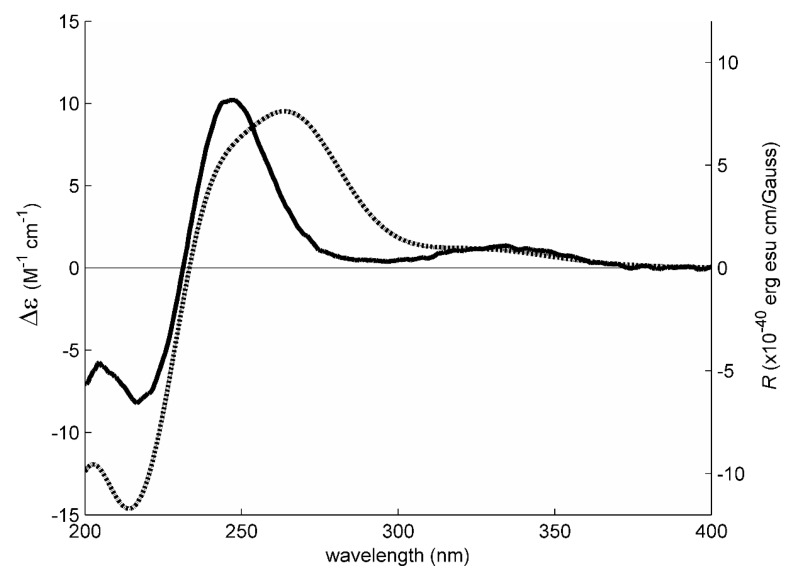
Experimental electronic circular dichroism (ECD) spectrum (solid line, left axis) of **1** in methanol and theoretical ECD spectrum (dotted line, right axis) of its (3*S*, 5*R*, 10*S*) configuration.

Structurally, **1** possesses three stereogenic carbons, i.e., C-3, C-5 and C-10. Since **1** was obtained as a viscous oil, it was not possible to obtain a suitable crystal to determine the absolute configurations of its stereogenic carbons by X-ray analysis. On the other hand, since **1** has an enone chromophore, the absolute configurations of its stereogenic carbons were established by comparison of the calculated and experimental electronic circular dichroism (ECD) spectra. Compound **1** has three stereogenic centers and, therefore, eight possible configurations. Conformational analysis revealed 36 molecular mechanics (MM) conformational energy minima for each configuration, by combining two conformations for each ring and three conformations for each of the two hydroxyl groups. To find the most significant conformations of each configuration, MM models were re-minimized using a quantum mechanical density functional theory (DFT) method coupled with a small basis set. Then, the most populated (lowest energy) conformations, spanning a window of 2 kcal/mol, were further refined using the same DFT method but with a large basis set. The first 50 ECD transitions were then calculated (TD-DFT) for each model. Comparison of the experimental ECD spectrum with the eight final calculated spectra, one for each configuration, revealed a better match with the spectrum obtained from the (3*S*, 5*R*, 10*S*) model (Figure 3 and Figure 4).

**Figure 5:**
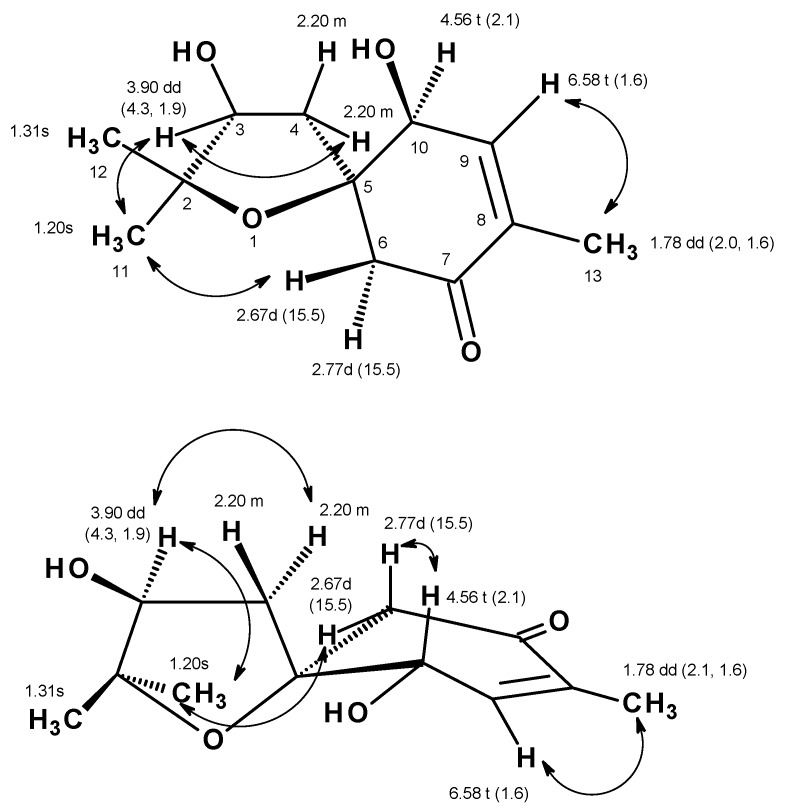
Key ROESY correlations in **1**.

The structure of **1** can be considered as an analogue of acremine O (compound **6** in Ref. [10]), where C-4 is oxidized to a ketone carbonyl and C-5 is chlorinated (according to the numbering in Ref. [10]). Although the absolute configuration of C-5 in **1** is the same as that of acremine O (C-3 in Ref. [10]), the absolute configuration of C-3 in **1** is opposite to that of acremine O (C-8 in Ref. [10]). Intrigued by this discrepancy, we obtained the ROESY spectrum of **1** and compared its data to what would be expected from the energy-optimized conformations of the (3*S*, 5*R*, 10*S*) and (3*R*, 5*R*, 10*S*) DFT models of **1**. The ROESY spectrum (Figures S8 and S9) exhibited correlations from H-3 to H_2_-4 and H_3_-11, whereas H_3_-11 exhibited correlations not only to H_2_-4 but also to H-6 at δ_H_ 2.67 (d, *J* = 15.5 Hz). These ROESY correlations implied that H-3, Me-11 and H-6 (at δ_H_ 2.67) are on the same face. That means OH-3 and Me-12 are on the opposite face to Me-11 and H-3. On the other hand, H-10 exhibited a weak correlation to H-6 at δ_H_ 2.77 (d, *J* = 15.5 Hz), suggesting that they were on the same face. The difference in the chemical shift values of H_2_-6 was attributed to the anisotropic effect of the carbonyl group (C-7) of the cyclohexanone ring. One of H_2_-6 (δ_H_ 2.67, d, *J* = 15.5 Hz) was deshielded when compared to another (δ_H_ 2.77, d, *J* = 15.5 Hz), implying that the former was in the equatorial position (H-6β) while the latter was in the axial position (H-6α) (Figure 5). 

These ROESY correlations are compatible with the (3*S*, 5*R*, 10*S*) model because, in this model, all these nuclei are on the same side of the five-membered ring and near enough to observe NOE signals (Figure 3). Inversion of the configuration of C-3 from (*S*) to (*R*) puts H-3 and H-6 (δ_H_ 2.77, d, *J* = 15.5 Hz) on opposite ringsides (and a bit further from the C-2 methyl groups). Therefore, according to our C-3(*R*) configuration model, H-3 and H-6 (δ_H_ 2.67, d, *J* = 15.5 Hz) are expected to show (probably weak) NOE cross peaks to both Me-11 and Me-12. Since H-3 and H-6 (δ_H_ 2.67, d, *J* = 15.5 Hz) are both observed to be near Me-11 and not Me-12, as inferred from ROESY data, we propose the (3*S*, 5*R*, 10*S*) configurations for **1**. Since **1** has never been previously reported, it was named acremine S, following the series of the prenylated polyketides, isolated from *Acremonium* species.

**Figure 6:**
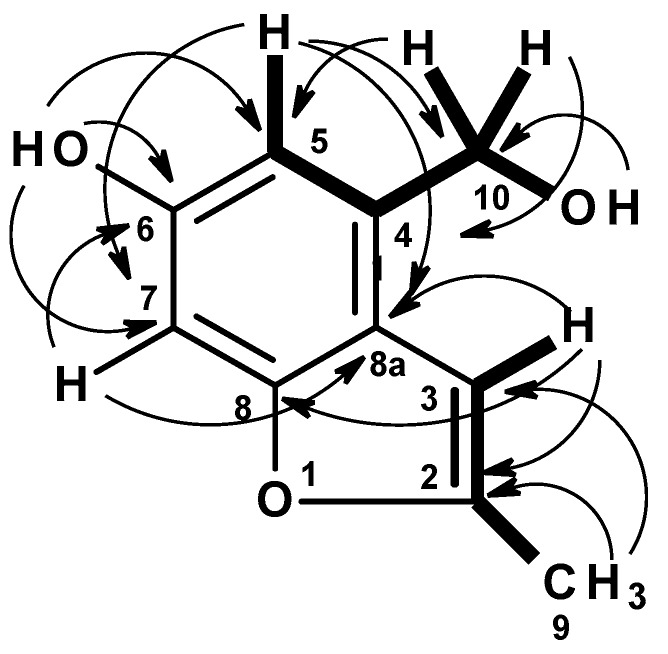
Key COSY (bold line) and HMBC (arrow) correlations in **2**.

Compound **2** was isolated as a yellow amorphous solid. The ^1^H NMR in DMSO-d_6_ (Table 1, Figure S11) spectrum showed that it still contained some impurities. However, the major component displayed a broad singlet of a phenolic hydroxyl proton at δ_H_ 9.28, a singlet of two aromatic proton at δ_H_ 6.70, a broad singlet of an olefinic proton at δ_H_ 6.47, a triplet at δ_H_ 5.17 (*J* = 5.6 Hz, 1H), a doublet at δ_H_ 4.58 (*J* = 5.6 Hz, 2H), a methyl doublet at δ_H_ 2.36 (*J* = 1.0 Hz). The ^13^C NMR (Table 2, Figure S12) exhibited ten carbon signals which were categorized, by DEPT and HSQC spectra (Table 2, Figures S13, S14 and S16), as five non-protonated sp^2^ (δ_C_ 155.0, 154.4, 152.6, 134.7 and 118.4), three protonated sp^2^ (δ_C_ 109.3, 101.0 and 95.6), one oxymethylene sp^3^ (δ_C_ 61.0) and one methyl (δ_C_ 13.7) carbons. The COSY spectrum (Table 2, Figure 6, Figure S15) displayed correlations from the singlet at δ_H_ 6.70 (H-5) to the doublet at δ_H_ 4.58 (H_2_-10), from the hydroxyl triplet at δ_H_ 5.17 (OH-10) to H_2_-10. The presence of a 2,3-substituted 5-hydroxybenzyl alcohol was substantiated by HMBC correlations (Table 2, Figure 6, Figure S17) from a broad singlet of the phenolic hydroxyl proton (OH-6) to C-5 (δ_C_ 109.3/δ_H_ 6.70), C-7 (δ_C_ 95.6/δ_H_ 6.70), C-6 (δ_C_ 154.4, weak), H-5 to C-7, C-8a (δ_C_ 118.4), C-6, and C-10 (δ_C_ 61.0/δ_H_ 4.58), H-10 to C-5, C-8a, C-4 (δ_C_ 134.7), and OH-10 (δ_H_ 5.17, t, *J* = 5.6 Hz) to C-10.

That the 2,3-substituted 5-hydroxybenzyl alcohol was fused to a 2-methylfuran ring, through C-8 (δ_C_ 155.0) and C-8a was supported by HMBC correlations (Table 2, Figure S16, Figure 6) from H-3 (δ_H_ 6.47, brs) to C-8a, C-8, C-2 (δ_C_ 152.6), and from the methyl doublet at δ_H_ 2.36 (CH_3_-9; δ_C_ 13.7) to C-2 and C-3 (δ_C_ 101.0).

In order to confirm the structure of **2**, the compound was further purified by passing through a Sephadex LH-20 column, and its ^1^H and ^13^C NMR spectra (Figures S18 and S19) showed that the impurities were completely removed. Interestingly, the ^1^H NMR spectrum of the pure compound, also in DMSO-d_6_, did not exhibit the triplet of OH-10 (δ_H_ 5.17, *J* = 5.6 Hz) while H_2_-10 appeared as a singlet at δ_H_ 4.58 instead of as a doublet with *J* = 5.6 Hz. The HSQC and HMBC spectra (Figures S20 and S21) were also in agreement with those obtained before re-purification. Since the pure compound exhibited the (–)-HRESIMS *m/z* at 177. 0556 (M − H)^+^ (calculated for C_10_H_9_O_3_, 177.0552) (Figure S22), its molecular formula was established as C_10_H_10_O_3_, corresponding to six degrees of unsaturation. Therefore, **2** was elucidated as 4-(hydroxymethyl)-2-methyl-1-benzofuran-6-ol. A structure search through SciFinder displayed the structure of **2** with the name 6-hydroxy-2-methyl-4-benzofuranmethanol and the CAS Registry Number 1083198-52-0. However, there is no reference reporting its source or its NMR data. Therefore, **2** was named acremine T. 

Compounds **1**–**3** were assayed for their *in vitro* inhibitory activities against AChE and BuChE, and the results are shown in Table 3 and Table 4. Lumichrome (**3**) was found to exhibit AChE inhibitory capacity (IC_50_ = 12.24 ± 0.12) comparable to that of galantamine (IC_50_ = 11.31 ± 0.11), while acremines S (**1**) and T (**2**), at a concentration of 6.6 µM, showed a much weaker inhibition than lumichrome (**3**). On the contrary, acremine S (**1**) showed inhibitory activity against BuChE three folds higher than that of galantamine, whereas acremine T (**2**) exhibited comparable activity to that of galantamine, and lumichrome (**3**) showed weak activity against BuChE at a concentration of 6.25 µM.

## 3. Experimental Section

### 3.1. General Experimental Procedures

The melting points were determined on a Stuart Melting Point Apparatus SMP3 (Bibby Sterilin, Stone, Staffordshire, UK) and were uncorrected. Optical rotations were measured on an ADP410 Polarimeter (Bellingham + Stanley Ltd., Tunbridge Wells, Kent, UK). ^1^H and ^13^C NMR spectra were recorded at ambient temperature on a Bruker AMC instrument (Bruker Biosciences Corporation, Billerica, MA, USA) operating at 300 or 500 and 75 or 125 MHz, respectively. High resolution mass spectra were measured with a Waters Xevo QToF mass spectrometer (Waters Corporations, Milford, MA, USA) coupled to a Waters Aquity UPLC system. A Merck (Darmstadt, Germany) silica gel GF_254_ was used for preparative TLC, and a Merck Si gel 60 (0.2–0.5 mm) was used for column chromatography.

### 3.2. Fungal Material

The fungus was isolated from the marine sponge *Mycale* sp. which was collected, by scuba diving at a depth of 5–10 m, from the coral reef at Samaesan Island (12°34′36.64″ N, 100°56′59.69″ E), in the Gulf of Thailand, in September 2016. The sponge was washed with 0.01% sodium hypochlorite solution for 1 min, followed by sterilized seawater three times, and then dried on sterile filter paper under sterile aseptic conditions. The sponge was cut into small pieces (5 mm × 5 mm) and placed on Petri dish plates containing 15 mL potato dextrose agar (PDA) medium mixed with 300 mg/L of streptomycin sulfate and incubated at 28 °C for 7 days. The hyphal tips emerging from sponge pieces were individually transferred onto PDA slants and maintained as pure cultures at Kasetsart University Fungal Collection, Department of Plant Pathology, Faculty of Agriculture, Kasetsart University, Bangkok, Thailand, for further identification. The fungal strain KUFA 1007 was identified as *Acremonium persicinum*, based on morphological characteristics such as colony growth rate and growth pattern on standard media, namely Czapek’s agar, Czapek yeast autolysate agar and malt extract agar. Microscopic characteristics including size, shape and ornamentation of conidiophores and spores were examined under light and scanning electron microscopes. This identification was confirmed by molecular techniques using internal transcribed spacer (ITS) primers. DNA was extracted from young mycelia following a modified Murray and Thompson method [22]. Primer pairs ITS1 and ITS4 [23] were used for ITS gene amplification. PCR reactions were conducted on Thermal Cycler and the amplification process consisted of the initial denaturation at 95 °C for 5 min, 34 cycles at 95 °C for 1 min (denaturation), at 55 °C for 1 min (annealing) and at 72 °C for 1.5 min (extension), followed by final extension at 72 °C for 10 min. The PCR products were examined by agarose gel electrophoresis (1% agarose with 1 × Tris-Borate-EDTA (TBE) buffer) and visualized under UV light after staining with ethidium bromide. DNA sequencing analyses were performed using the dideoxyribonucleotide chain termination method [24] by Macrogen Inc. (Seoul, Korea). The DNA sequences were edited using the FinchTV software (version 1.4, Geospiza Inc, Seattle, WA, USA) and submitted into the BLAST program for alignment and compared to fungal species in the NCBI database (http://www.ncbi.nlm.nih.gov/). Its gene sequences were deposited in GenBank with accession number MG755248.

### 3.3. Extraction and Isolation

The fungus was cultured for one week at 28 °C in five Petri dishes (i.d. 90 mm) containing 20 mL of potato dextrose agar per dish. The mycelial plugs (5 mm in diameter) were transferred to two 500 mL Erlenmeyer flasks containing 200 mL of potato dextrose broth and incubated on a rotary shaker at 120 rpm at 28 °C for one week. Fifty 1000 mL Erlenmeyer flasks, each containing 300 g of cooked rice, were autoclaved at 121 °C for 15 min. After cooling to room temperature, 20 mL of a mycelial suspension of the fungus was inoculated per flask and incubated at 28 °C for 30 days, after which 500 mL of EtOAc was added to each flask of the moldy rice and macerated for 7 days, and then filtered with Whatman No. 1 filter paper (GE Healthcare UK Limited, Buckinghamshire, UK). The EtOAc solutions were combined and concentrated under reduced pressure to yield 59.8 g of crude EtOAc extract which was dissolved in 500 mL of EtOAc and then filtered with Whatman No. 1 filter paper. The EtOAc solution was then washed with H_2_O (3 × 500 mL) and dried with anhydrous Na_2_SO_4_, filtered and evaporated under reduced pressure, to give 46.3 g of crude EtOAc extract. The crude EtOAc extract was applied on a column chromatography of silica gel (390 g), and eluted with mixtures of petrol–CHCl_3_ and CHCl_3_–Me_2_CO, wherein 250 mL fractions (Frs) were collected as follows: Frs 1–23 (petrol–CHCl_3_, 1:1), 24–49 (petrol–CHCl_3_, 3:7), 50–177 (petrol–CHCl_3_, 1:9), 178–207 (CHCl_3_), 208–453 (CHCl_3_–Me_2_CO, 9:1), 454–546 (CHCl_3_–Me_2_CO, 7:3). Frs 54–75 were combined (296.5 mg) and recrystallized in MeOH to give **4** (125.3 mg). Fr 213 (264.3 mg) was crystallized in MeOH to give **5** (48.0 mg). Frs 214–246 were combined (2.10 g) and applied on a column chromatography of silica gel (40 g) and eluted with mixtures of petrol–CHCl_3_ and CHCl_3_–Me_2_CO, wherein 100 mL sub-fractions (sfrs) were collected as follows: sfrs 1–52 (petrol–CHCl_3_, 3:7), 53–74 (petrol–CHCl_3_, 1:9), 75–158 (CHCl_3_), 159–218 (CHCl_3_: Me_2_CO, 19:1), 219–254 (CHCl_3_: Me_2_CO, 1:9). Sfrs 122–160 were combined (314.4 mg) and recrystallized in MeOH to give **5** (60.2 mg). Frs 246–255 were combined (174.7 mg) and purified by TLC (silica gel G_254_, CHCl_3_:Me_2_CO:HCO_2_H, 8:2:0.1) to give 10.4 mg of **2** (with impurities), which was further applied on a Sephadex LH-20 column (5 g) and eluted with MeOH, wherein 67 sfrs of 1 mL were collected. Sfrs 40–50 were combined and, after evaporation of solvent, gave 2.4 mg of **2** as a pure compound. The other part of the TLC separation of frs 246–255 was combined with frs 256–285 (318.1 mg) and applied on a column chromatography of silica gel (30 g) and eluted with mixtures of petrol–CHCl_3_ and CHCl_3_–Me_2_CO, wherein 100 mL sfrs were collected as follows: Sfrs 1–63 (petrol–CHCl_3_, 3:7), 64–127 (CHCl_3_: Me_2_CO, 9:1) and 128–161 (CHCl_3_: Me_2_CO, 7:3). Sfrs 71–75 were combined (12.0 mg) and purified by TLC (silica gel G_254_, CHCl_3_:Me_2_CO:HCO_2_H, 8:2:0.1) to give 30.9 mg of a compound which was applied on a Sephadex LH-20 column (5 g) and eluted with a 1:1 mixture of CH_2_Cl_2_:MeOH, wherein 62 sfrs of 1 mL were collected. By using TLC to monitor the profile of eluted compounds, these sub-fractions were divided in two groups: Group I (sfrs 8–18) and group II (sfrs 19–30). Sfrs 8–18 (group I) were combined (19.7 mg), and applied on a Sephadex LH-20 column (5 g) and eluted with a 1:1 mixture of CH_2_Cl_2_: MeOH, wherein 40 sub-sfrs (ssfrs) of 1 mL were collected. Ssfrs 15–36 were combined and, after evaporation of solvent, gave 15.0 mg of **1**. Sfrs 19–30 (group II) were combined (5.1 mg), and applied on a Sephadex LH-20 column (5 g) and eluted with MeOH, wherein 20 ssfrs of 1 mL were collected. Sfrs 5–15 were combined and, after evaporation of solvent, gave 2.3 mg of **2**. Frs 286–315 were combined (189.3 mg) and crystallized in Me_2_CO to give 6.0 mg of **3**. The mother liquor was combined with frs 316–365 (291.9 mg) and applied on a column chromatography of silica gel (10 g), and eluted with mixtures of petrol–CHCl_3_ and CHCl_3_–Me_2_CO, wherein 100 mL sfrs were collected as follows: sfrs 1–22 (petrol:CHCl_3_, 1:1), 23–41 (petrol:CHCl_3_, 7:3), 54–73 (CHCl_3_), 74–162 (CHCl_3_: Me_2_CO, 19:1), 163–178 (CHCl_3_: Me_2_CO, 9:1). Sfrs 79–90 were combined (98.8 mg) and recrystallized in MeOH to give an additional 20.1 mg of **3**. 

#### 3.3.1. Acremine S [(3S, 5R, 10S)-3,10-dihydroxy-2,2,8-trimethyl-1-oxospiro[4.5]dec-8-en-7-one] (**1**) 

Yellowish viscous oil; [α]${}_{20}^{D}$ +44 (c 0.5, CHCl_3_). For ^1^H and ^13^C NMR spectroscopic data (CDCl_3_, 300 and 75 MHz), see Table 1; (+)-HRESIMS *m*/*z* 227.1293 [M + H]^+^ (calculated for C_12_H1_9_O_4_, 227.1283).

#### 3.3.2. Acremine T [4-(hydroxymethyl)-2-methyl-1-benzofuran-6-ol] (**2**)

White amorphous solid. For ^1^H and ^13^C NMR spectroscopic data (CDCl_3_, 300 and 75 MHz), see Table 2; (–)-HRESIMS *m*/*z* 177.0556 [M − H]^+^ (calculated for C_10_H1_9_O_3_, 177.0552).

### 3.4. Electronic Circular Dichroism (ECD) of 1

The ECD spectrum of **1** (7 mM in methanol) was obtained in a Jasco J-815 CD spectropolarimeter (Jasco, Mary’s Court, Easton, MD, USA) with a 0.1 mm cuvette and eight accumulations. A conformational search, including dihedral driver and molecular mechanics MM2 minimizations, was done in Chem3D Ultra (Perkin-Elmer Inc., Waltham, MA, USA). Quantum chemical DFT minimizations were performed with Gaussian 16W (Gaussian Inc., Wallingford, CT, USA) using the APFD method with a 6-31G basis set, for a pre-selection of the most stable conformations, and a 6-311+G(2d,p) basis set for final minimizations and ECD spectral calculations (TD-DFT, 50 transitions). All DFT calculations included an IEFPCM solvation model of methanol. The spectrum of each relevant configuration/conformation was constructed by applying a Gaussian line broadening of 0.3 eV to each computed transition; no UV-shift was applied. The final theoretical spectrum of each possible configuration of **1** was obtained by a Boltzmann-weighted sum of the ECD spectra of its most abundant conformations, accounting for at least 90% of the theoretical conformer population [25].

### 3.5. Acetylcholinesterase (AChE) and Butyrylcholinesterase (BuChE) Assays

Acetylcholinesterase inhibitory activity was determined spectroscopically using the Ellman’s microplate method, with modification [26]. Briefly, 20 µL of 0.22 U/mL AChE of *Electrophorus electricus* (EC 3.1.1.7, Sigma-Aldrich, Saint Louis, MO, USA) was added to each well containing 10 µL of tested compound (200, 100, 50, 25, 12.5, 6.25, 3.125 and 1.563 µM in MeOH or DMSO), 100 µL of 3 mM of 5,5′-dithiobis(2-nitrobenzoic acid) (DTNB) and 20 µL of 15 mM acetylthiocholine iodide. Absorbance of the colored-end product was measured during 5 min, with 30 s intervals, at 412 nm, controlled by a BioTek Synergy™ HT Microplate Reader (Winooski, Vermont, USA). Controls containing 10 µL of compound vehicles (MeOH or DMSO) instead of tested compounds and blanks containing 20 µL of buffer [0.1% (*w/v*) bovine serum albumin in 50 mM Tris-HCl] instead of the enzyme were prepared. Percentage of enzyme inhibition was calculated as: % of inhibition = 100 − [((Abs_sample_-Abs_sample’s blank_)/(Abs_control_-Abs_blank_)) × 100] [27].

Each experiment was performed in triplicate, and galantamine was used as a positive control. The inhibitory activities of the compounds towards AChE were expressed as IC_50_, which was determined as the effective concentration at which AChE was inhibited by 50%. The IC_50_ was obtained by interpolation from linear regression analysis. When the solubilization of compounds was compromised, the IC_50_ could not be determined and, in this case, the percentage of inhibition for the maximum concentration tested was used.

For the butyrylcholinesterase inhibition assay, BuChE from lyophilized horse serum (EC 3.1.1.8, Merck, Damstadt, Germany) and butyrylthiochlorine iodide as a substrate for BuChE were used. All other reagents and conditions were the same except for 20 µL of the tested compounds were added per well.

### 3.6. Statistical Analysis

The assays were conducted in triplicate and all tabulated results were expressed as the mean ± SEM. Statistical analysis of the results was performed with GraphPad Prism (GraphPad Software, San Diego, CA, USA). An unpaired *t*-test was carried out to test for any significant difference between the means. Differences at the 5% confidence level were considered significant.

## 4. Conclusions

Marine-derived fungi are important sources of structurally diverse and biologically relevant secondary metabolites. Our group have investigated secondary metabolites from the marine-derived species of *Aspergillus*, *Neosartorya*, *Penicillium* and *Talaromyces* to evaluate their potential as antibiotics to combat multidrug-resistant pathogenic bacteria. Recently, we have focused our attention on naturally occurring compounds with potential for treatment of neurological disorders. Since only a few compounds from marine-derived fungi have been tested for neuroprotective activities, we have isolated secondary metabolites produced by marine-derived fungi to investigate their capacity as cholinesterase inhibitors. In this work, we described the isolation and structure elucidation of two previously undescribed secondary metabolites: acremines S (**1**) and T (**2**), in addition to three known compounds, including lumichrome (**3**), ergosterol (**4**) and ergosterol 5,8-endoperoxide (**5**), from the cultures of the fungus *Acremonium persicinum* strain KUFA1007, which was isolated from the marine sponge *Mycale* sp., collected in the Gulf of Thailand. The acetylcholinesterase and butyrylcholinesterase inhibitory activities of **1**–**3** were also evaluated. The results showed that lumichrome (**3**) possesses the inhibitory activity toward the enzyme acetylcholinesterase comparable to that of the standard drug galantamine, while acremine S (**1**) showed more potent antibutyrylcholinesterase activity than that of galantamine. The results of this study can pave the way to the exploitation of the secondary metabolites produced by cultures of marine-derived fungi for a development of the therapeutic arsenals for neurological disorders such as Alzheimer’s disease. 

## Figures and Tables

**Table 1:** ^1^H and ^13^C NMR (CDCl_3_, 300.13 and 75.4 MHz) and HMBC assignment for **1.**

Position	δ_C_, Type	δ_H_, (*J* in Hz)	COSY	HMBC
2	85.8. C	-	-	
3	77.3, CH	3.90, dd (4.3,1.9)	H-4	
4	39.4, CH_2_	2.20, m	H-3	C-2,3,5,6,10
5	87.1, C	-	-	
6a b	53.0, CH_2_	2.67, d (15.5) 2.77, d (15.5)	H-6b H-6a	C-4,5,7,8,10
7	197.8, CO	-	-	
8	135.6, C	-	-	
9	147.2, CH	6.58, t (1.6)	H-10, 13	C-5,7,13
10	73.6, CH	4.56, t (2.1)	H-9, 13	
11	27.8, CH_3_	1.20, s	-	C-2,3,12
12	22.6, CH_3_	1.31, s	-	C-2,8,11
13	15.0, CH_3_	1.78, dd (2.1,1.6)	H-9, 10	C-7,8,9

**Table 2:** ^1^H and ^13^C NMR (DMSO-d_6_, 300.13 and 75.4 MHz) and HMBC assignment for **2.**

Position	δ_C_, Type	δ_H_ (*J* in Hz)	COSY	HMBC
2	152.6, C	-		
3	101.0, CH	6.47, brs	CH_3_-9	C-2,4,8
4	134.7, C			
5	109.3, CH	6.70, s	H_2_-10	C-6,7,8a,10
6	154.4, C	-		
7	95.6, CH	6.70, s		C-6,8a
8	155.0, C	-		
8a	118.4, C	-		
9	13.7, CH_3_	2.36, d (1.0)		C-2,3
10	61.0, CH_2_	4.58, d (5.6)	H-5, OH-6	C-4,5,8a
OH-6	-	9.28, s		C-5,6,7
OH-10	-	5.17, t (5.6)	H_2_-10	C-10

**Table 3:** Acetylcholinesterase (AChE) inhibitory activity of **1**–**3**.

Compound	% inhibition at 6.6 µM	IC_50_ (µM)
**1**	10.42 ± 0.33 *	n.d.
**2**	14.08 ± 1.27 *	n.d.
**3**	39.23 ± 1.77 *	12.24 ± 0.12 **
**Galantamine**	39.76 ± 2.22 *	11.31 ± 0.11 **

Results are given as mean ± SEM of three independent experiments performed in triplicate; n.d. = not determined. * *p* < 0.05; ** *p* < 0.01.

**Table 4:** Butyrylcholine esterase (BuChE) inhibitory activity of **1**–**3**.

Compound	% inhibition at 6.25 µM	IC_50_ (µM)
**1**	30.71 ± 0.21 **	n.d.
**2**	10.53 ± 1.01 **	n.d.
**3**	3.44 ± 0.09 *	n.d.
**Galantamine**	10.34 ± 0.21 *	n.d.

Results are given as mean ± SEM of three independent experiments performed in triplicate; n.d. = not determined. * *p* < 0.05; ** *p* < 0.01.

## Supplementary Materials

The following are available online at https://www.mdpi.com/1660-3397/17/6/379/s1. Figures S1–S9, S11–S21, S23–S28 and S30–S33: 1D and 2D NMR spectra of compounds **1**–**5**. Figures S10, S22 and S29: HRMS data for compounds **1**–**3**. Table S1: ^1^H and ^13^C NMR (DMSO-d_6_, 500 and 125 MHz) for **3**.
