# Supplementary material for: A New Meroterpene, A New Benzofuran Derivative and Other Constituents from Cultures of the Marine Sponge-Associated Fungus Acremonium persicinum KUFA 1007 and Their Anticholinesterase Activities
Source: Mar Drugs. 2019 Jun 25;17(6):379. doi: 10.3390/md17060379 (PMC6628235; doi:10.3390/md17060379)
Supplement: Supplementary file 1 [file marinedrugs-17-00379-s001.pdf]

Supplementary Materials

A New Meroterpene, A New Benzofuran Derivative and Other Constituents from Cultures of the Marine Sponge-Associated Fungus *Acremonium persicinum* KUFA 1007 and Their Anticholinesterase Activities

Ana J. S. Alves [1, 2], José A. Pereira [2, 3], Tida Dethoup [4], Sara Cravo [1, 3], Sharad Mistry[5], Artur M. S. Silva [6], Madalena M. M. Pinto [3]* and Anake Kijjoa [2, 3]*

[1] Laboratório de Química Orgânica, Departamento de Ciências Químicas, Faculdade de Farmácia, Universidade do Porto, Rua de Jorge Viterbo Ferreira, 228, 4050-313 Porto, Portugal. E-mail: anajoao93@hotmail.com (A. J. S. A), scravo@ff.up.pt (S. C.)

[2] *ICBAS*-Instituto de Ciências Biomédicas Abel Salazar, Rua de Jorge Viterbo Ferreira, 228, 4050-313 Porto, Portugal. E-mail: jpereira@icbas.up.pt (J. A. P.)

[3] Interdisciplinary Centre of Marine and Environmental Research (CIIMAR), Terminal de Cruzeiros do Porto de Lexões, Av. General Norton de Matos s/n, 4450-208 Matosinhos, Portugal. E-mail: madalena@ff.up.pt.

[4] Department of Plant Pathology, Faculty of Agriculture, Kasetsart University, Bangkok 10240, Thailand. E-mail: tdethoup@yahoo.com.

[5] Department of Chemistry, University of Leicester, University Road, Leicester LE 7 RH, UK. E-mail: scm11@leicester.ac.uk.

[6] Departamento de Química & QOPNA, Universidade de Aveiro, 3810-193 Aveiro, Portugal. E-mail: artur.silva@ua.pt.

*Correspondence: ankijjoa@icbas.up.pt (A. K.); madalena@ff.up.pt (M. P.); Tel.: +351-22-042-8331 (A.K.) and +351-22-042-8331 (M. P.); Fax: +351-22-206-2232 (A.K.) and +351-22-206-2232 (M. P.)

Figure S1. ^{1}H NMR spectrum of **1** ($CDCl_3$, 300.13 MHz).

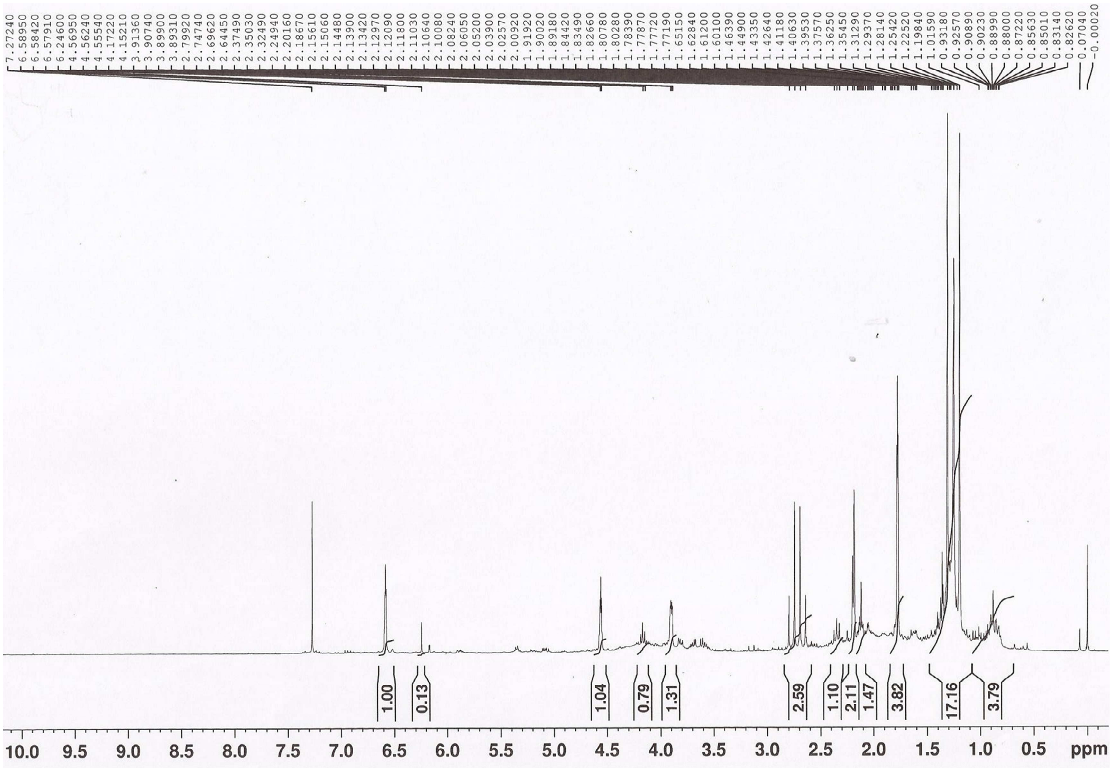

Figure S2. ^{13}C NMR spectrum of **1** ($CDCl_3$, 75.4 MHz).

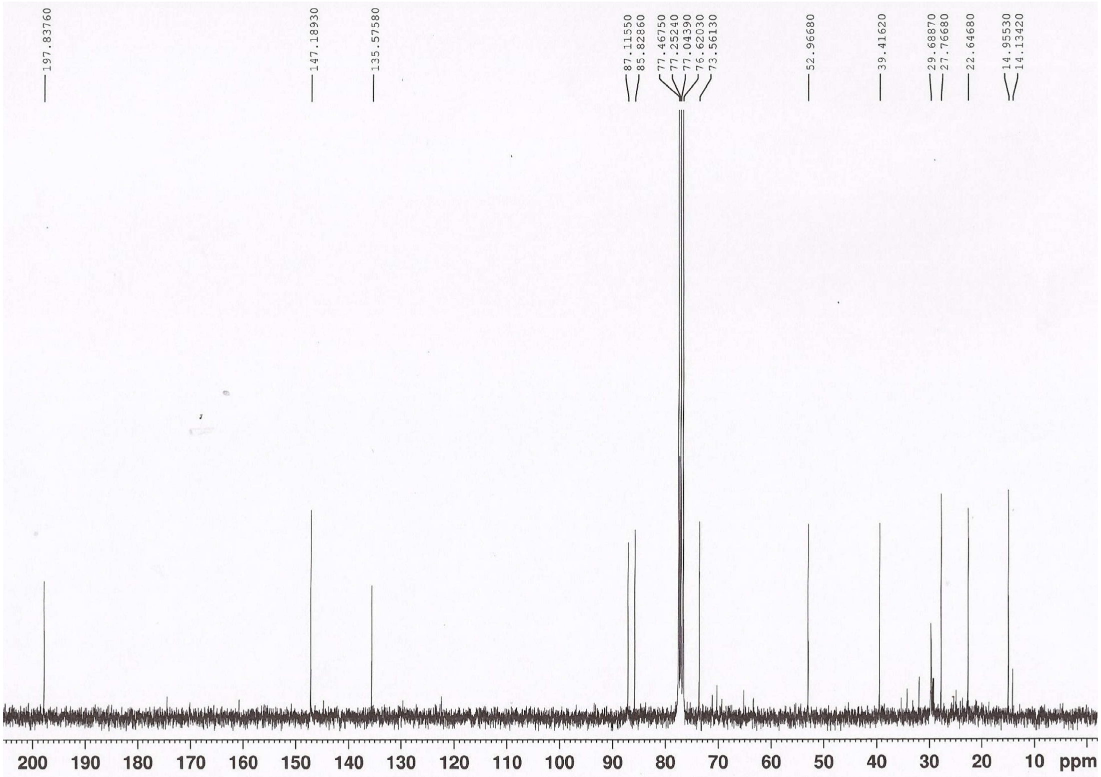

Figure S3. DEPT 135° spectrum of **1** ($CDCl_3$, 75.4 MHz).

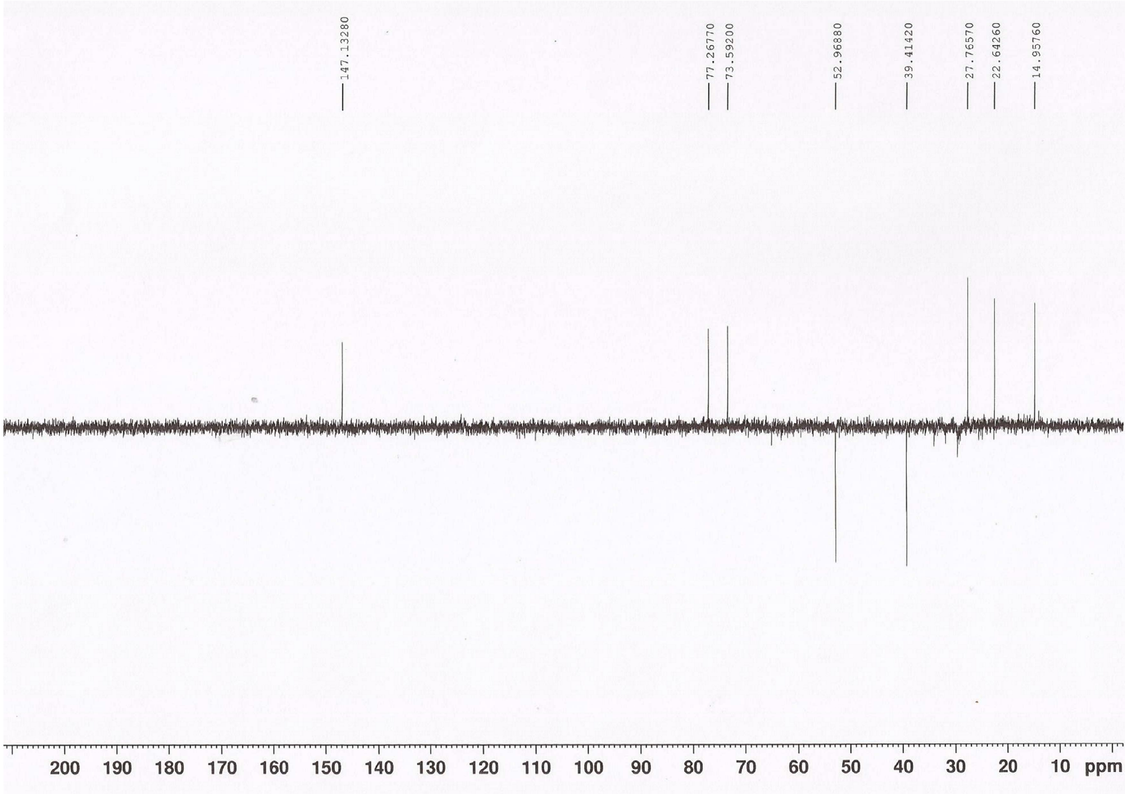

Figure S4. DEPT 90° spectrum of **1** ($CDCl_3$, 75.4 MHz).

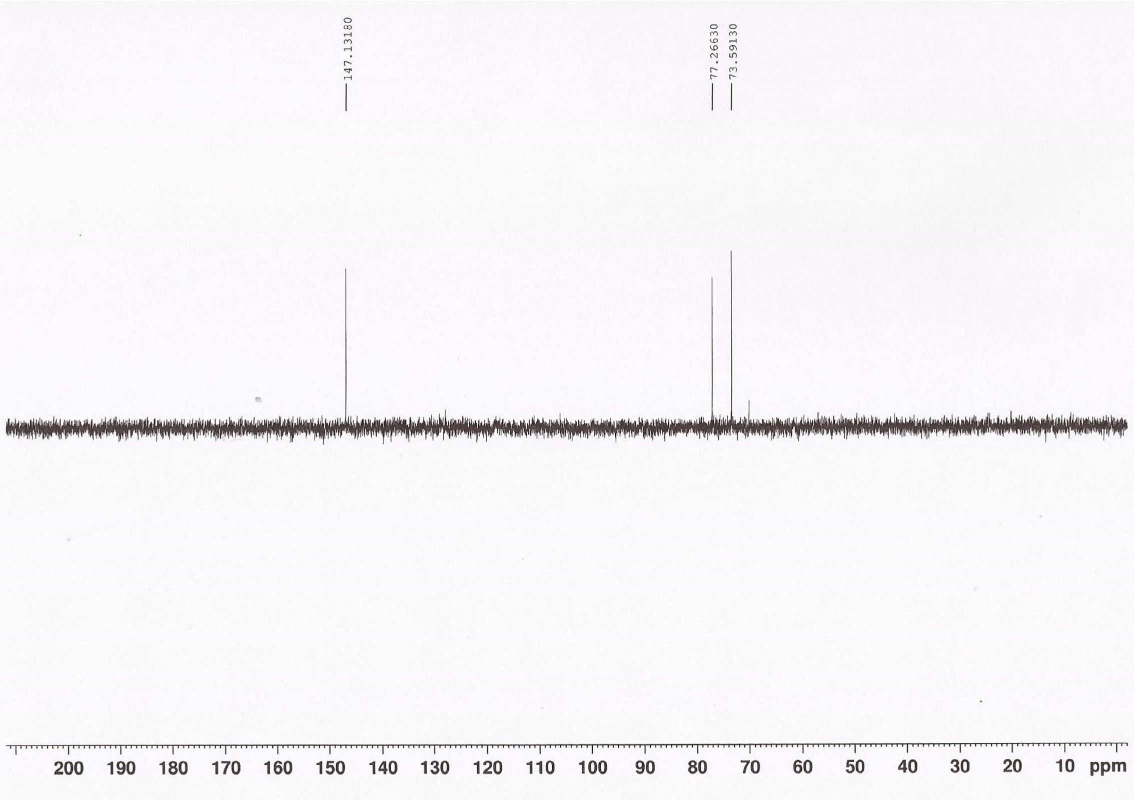

Figure S5. COSY spectrum of **1** ($CDCl_3$, 300.13 MHz).

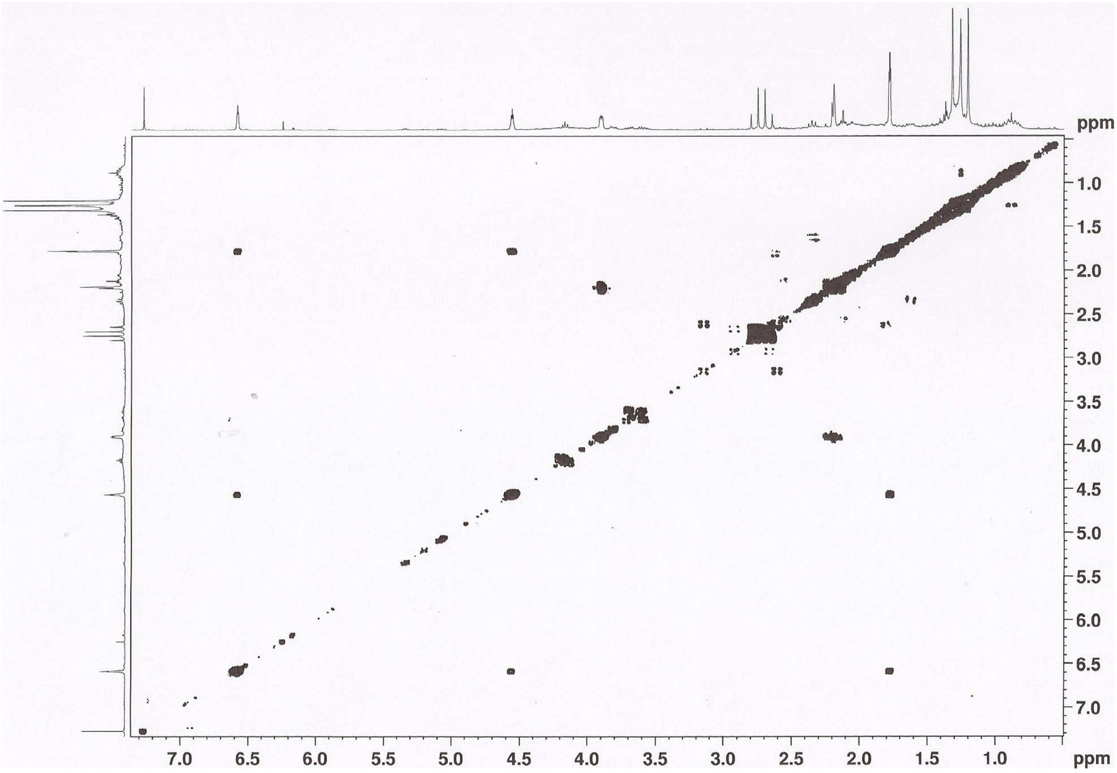

Figure S6. HSQC spectrum of **1** ($CDCl_3$, 300.13 MHz).

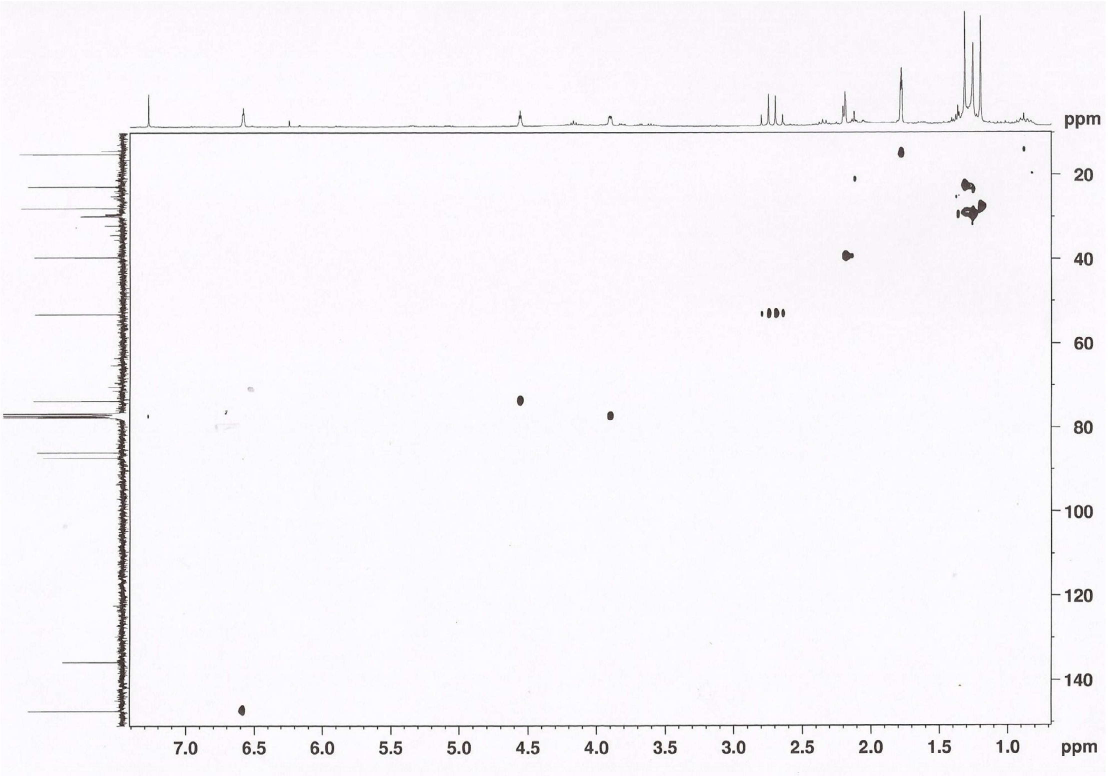

Figure S7. HMBC spectrum of **1** ($CDCl_3$, 300.13 MHz).

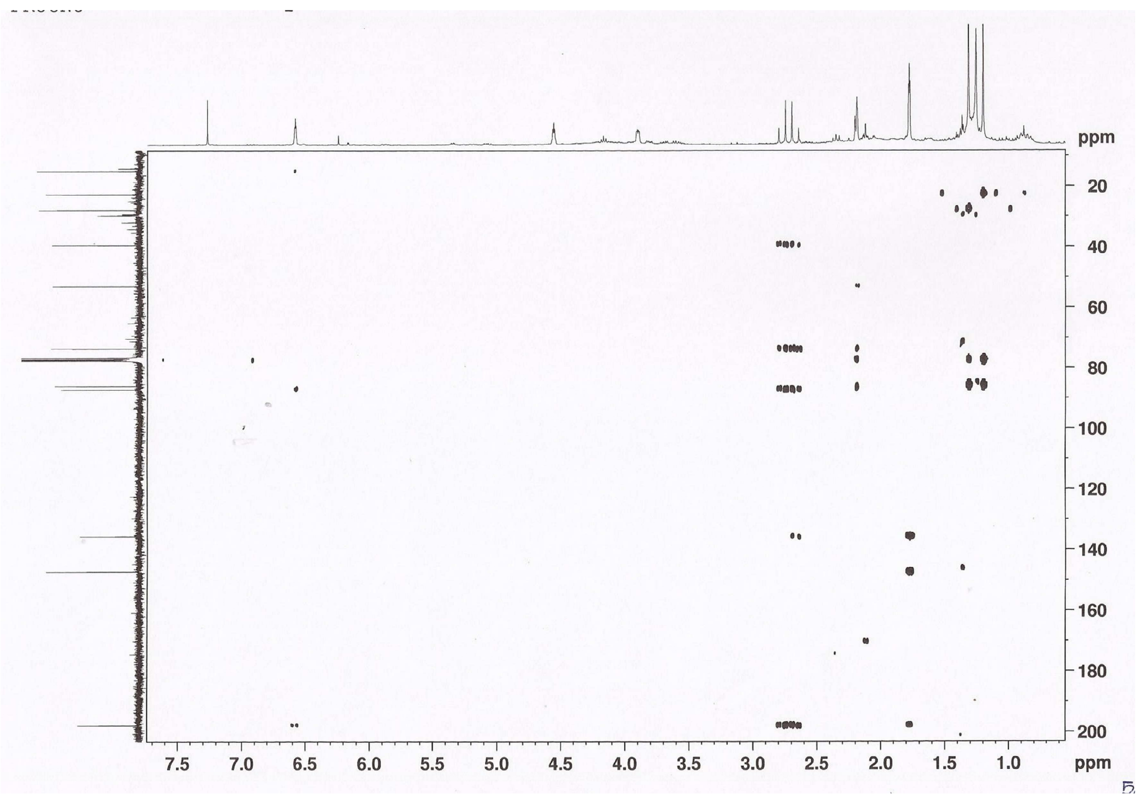

Figure S8. ROESY spectrum of **1** ($CDCl_3$, 500 MHz).

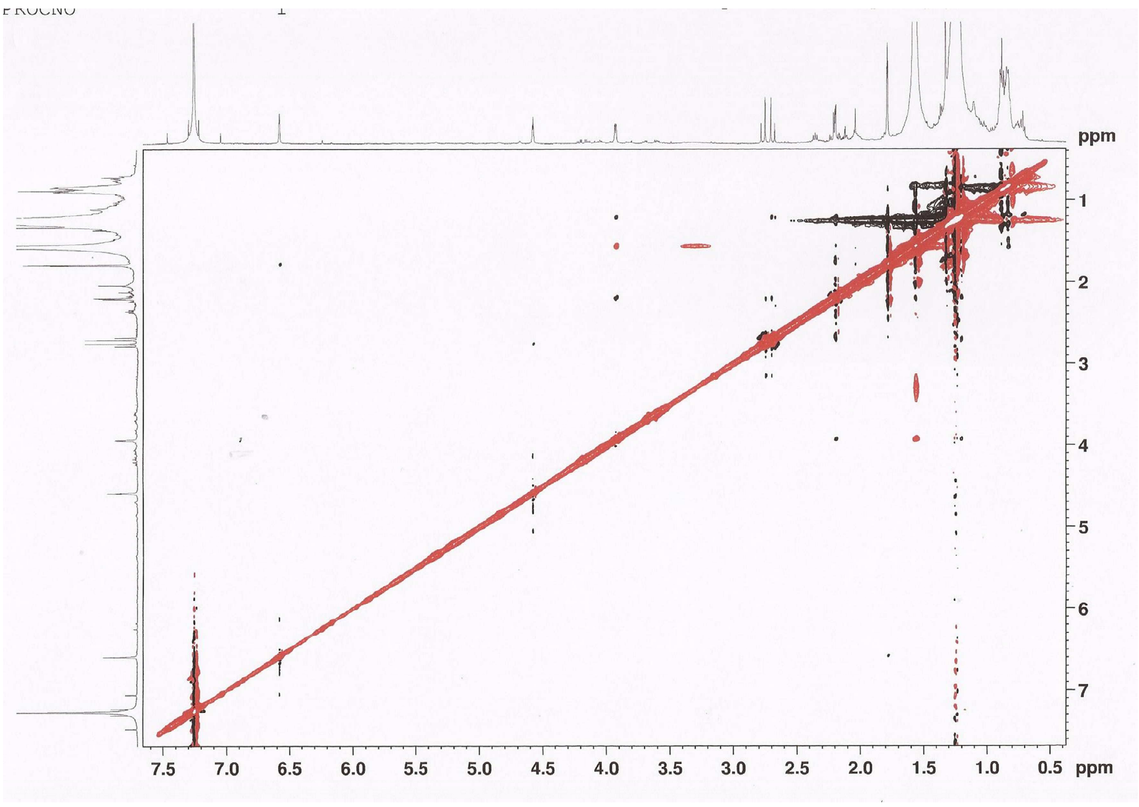

Figure S9. Expansion of ROESY spectrum of **1** ($CDCl_3$, 500 MHz).

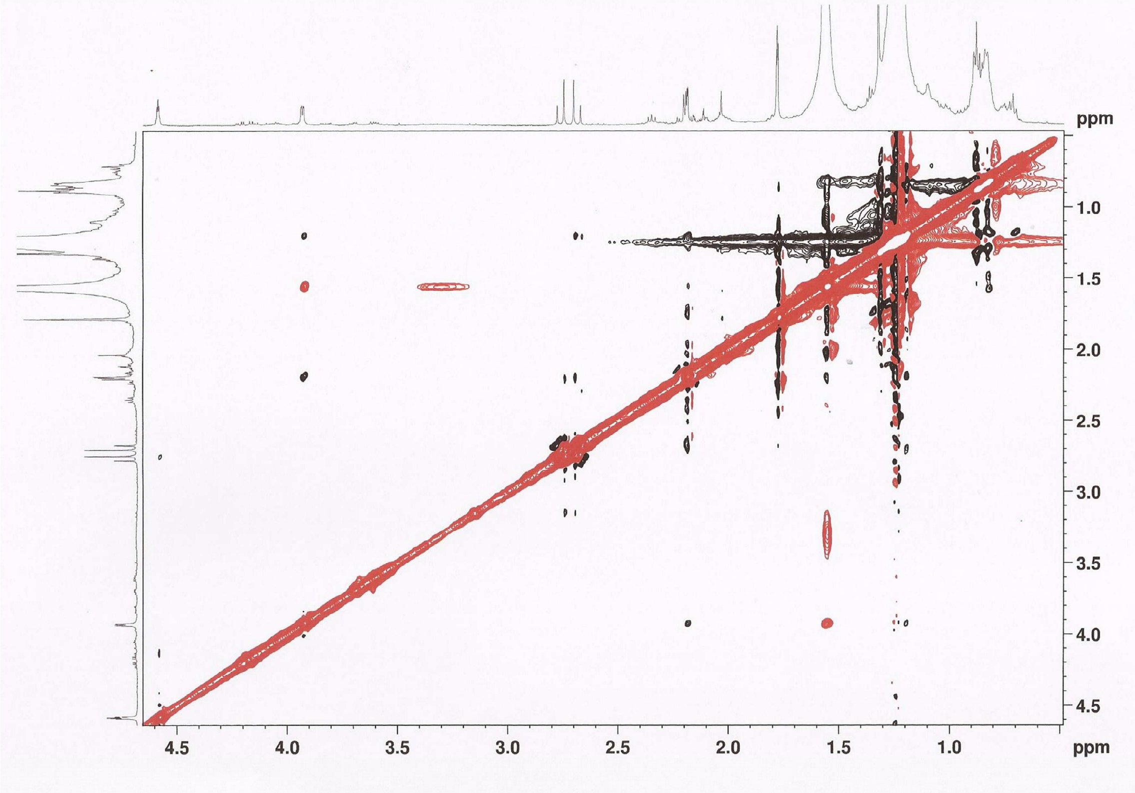

Figure S10. (+)-HRESIMS of **1.**

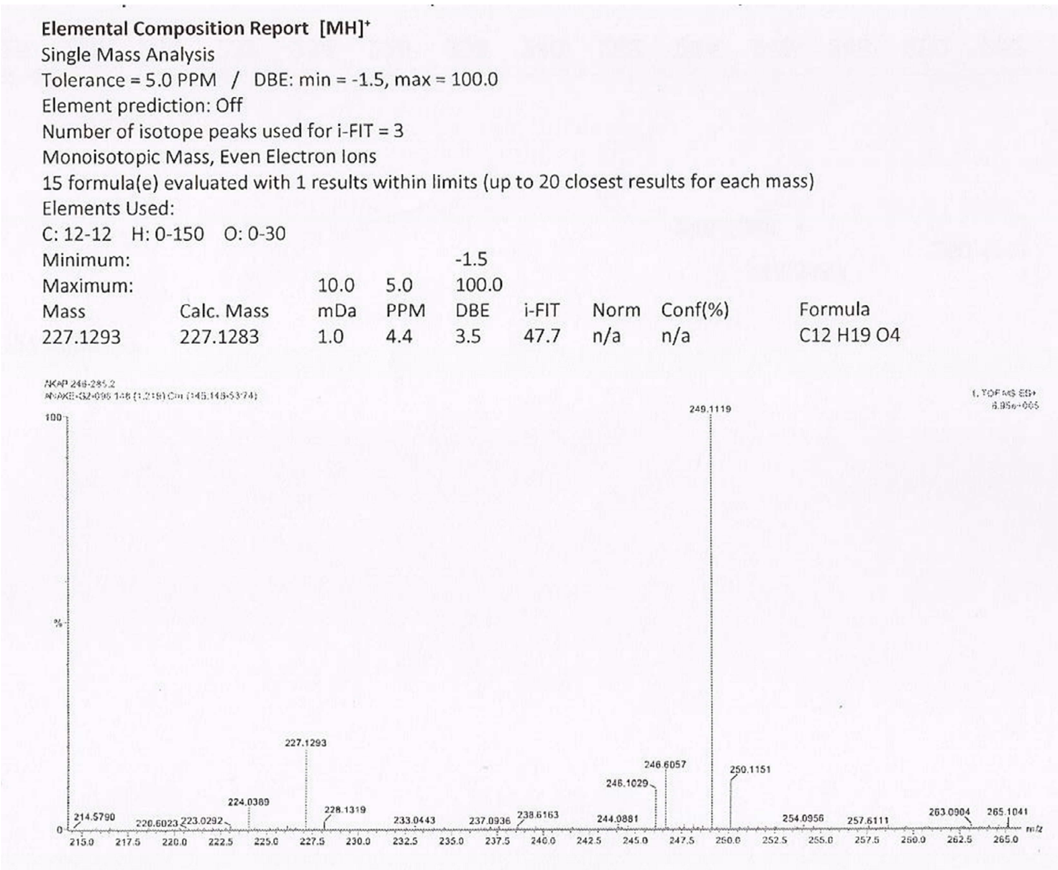

Elemental Composition Report [MH]+

Single Mass Analysis

Tolerance = 5.0 PPM / DBE: min = -1.5, max = 100.0

Element prediction: Off

Number of isotope peaks used for i-FIT = 3

Monoisotopic Mass, Even Electron Ions

15 formula(e) evaluated with 1 results within limits (up to 20 closest results for each mass)

Elements Used:

C: 12-12 H: 0-150 O: 0-30

Minimum:				-1.5				
Maximum:		10.0	5.0	100.0				
Mass	Calc. Mass	mDa	PPM	DBE	i-FIT	Norm	Conf(%)	Formula
227.1293	227.1283	1.0	4.4	3.5	47.7	n/a	n/a	C12 H19 O4

Figure S11. ^{1}H NMR spectrum of **2** (DMSO-d$_6$, 300.13 MHz) before purification.

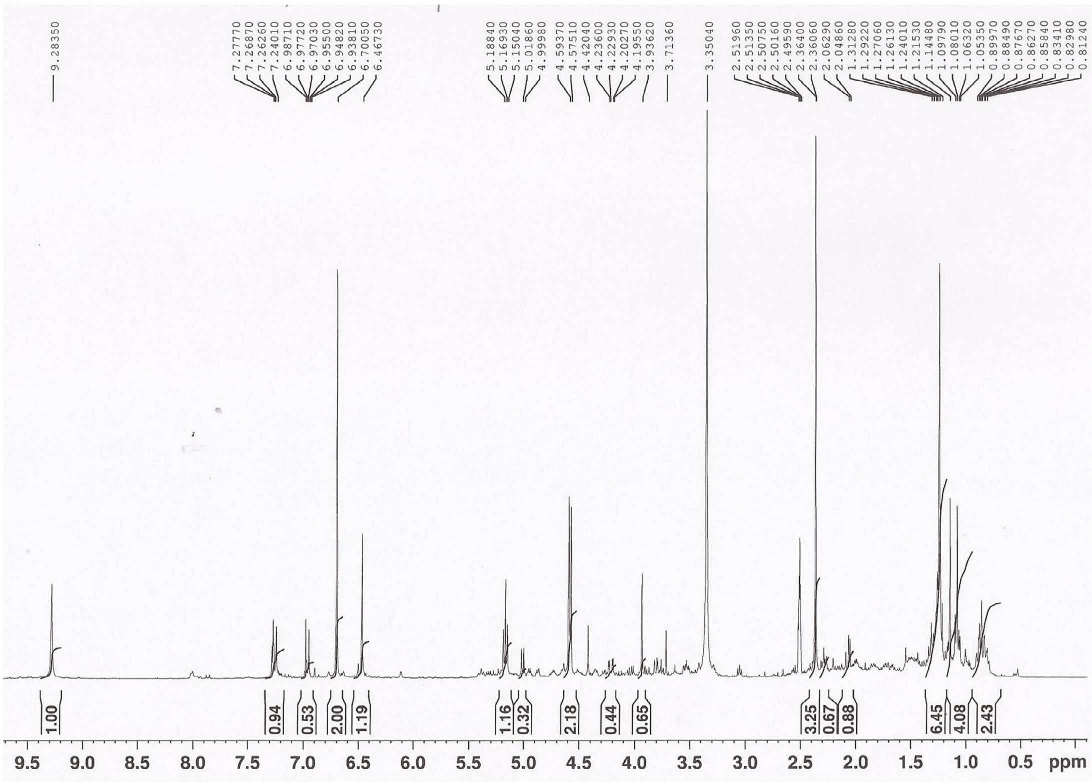

Figure S12. ^{13}C NMR spectrum of **2** (DMSO-d$_6$, 75.4 MHz) before purification.

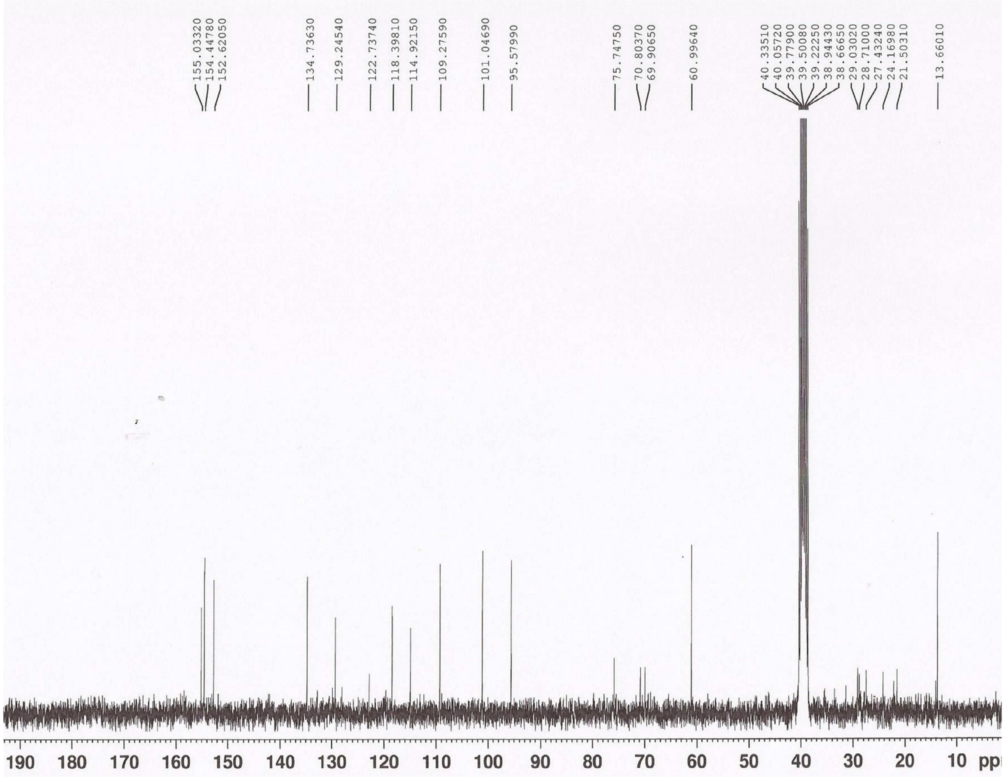

Figure S13. DEPT 135º spectrum of **2** (DMSO-d$_6$, 75.4 MHz) before purification.

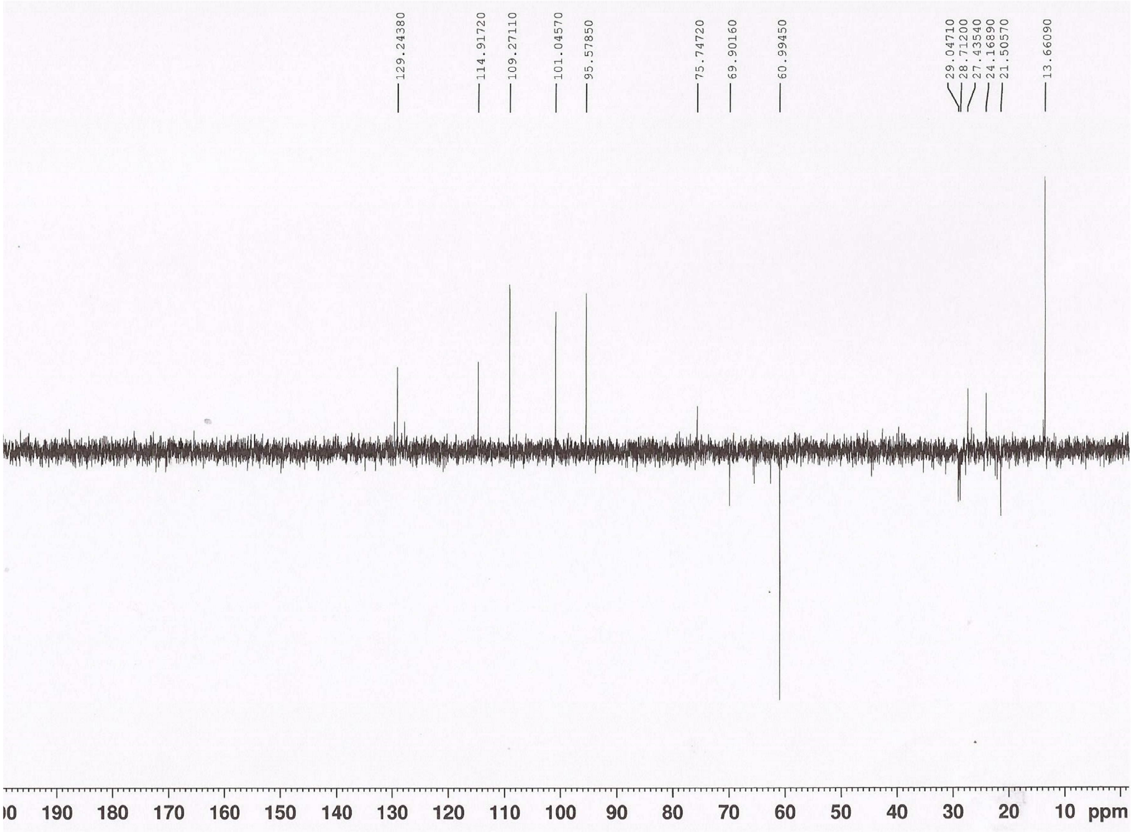

Figure S14. DEPT 90º spectrum of **2** (DMSO-d$_6$, 75.4 MHz) before purification.

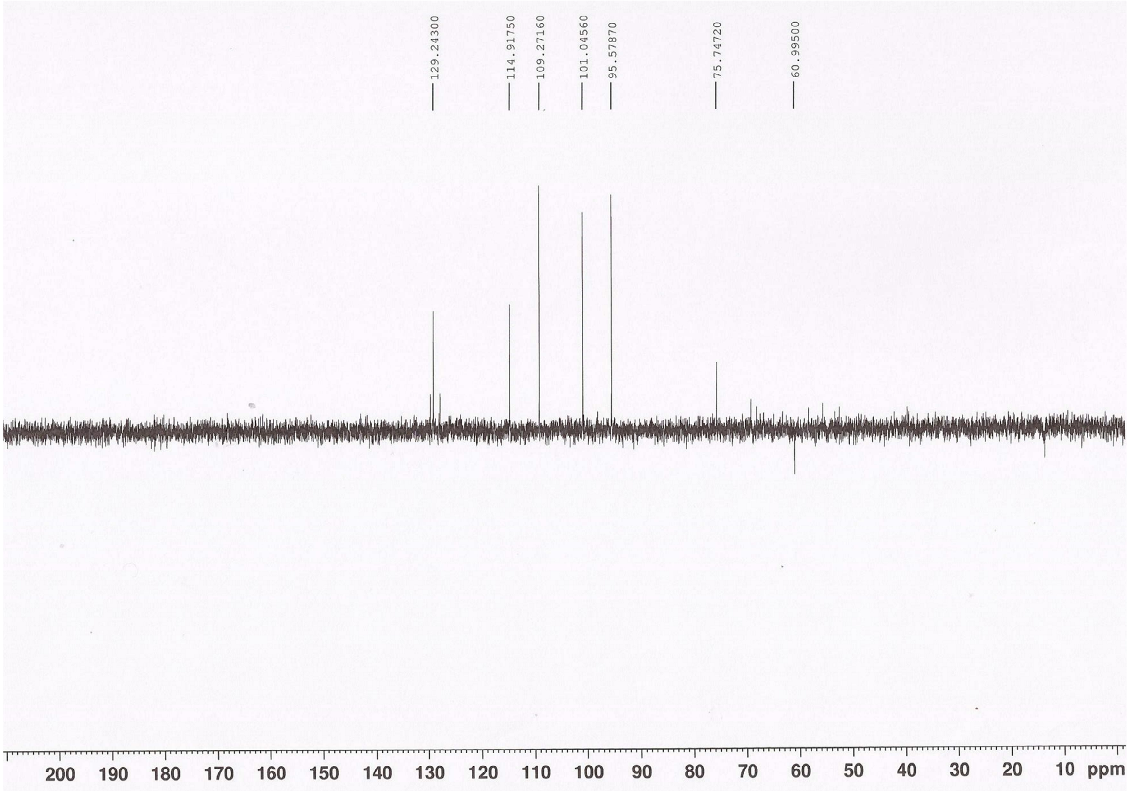

Figure S15. COSY spectrum of **2** (DMSO-d$_6$, 300.13 MHz) before purification.

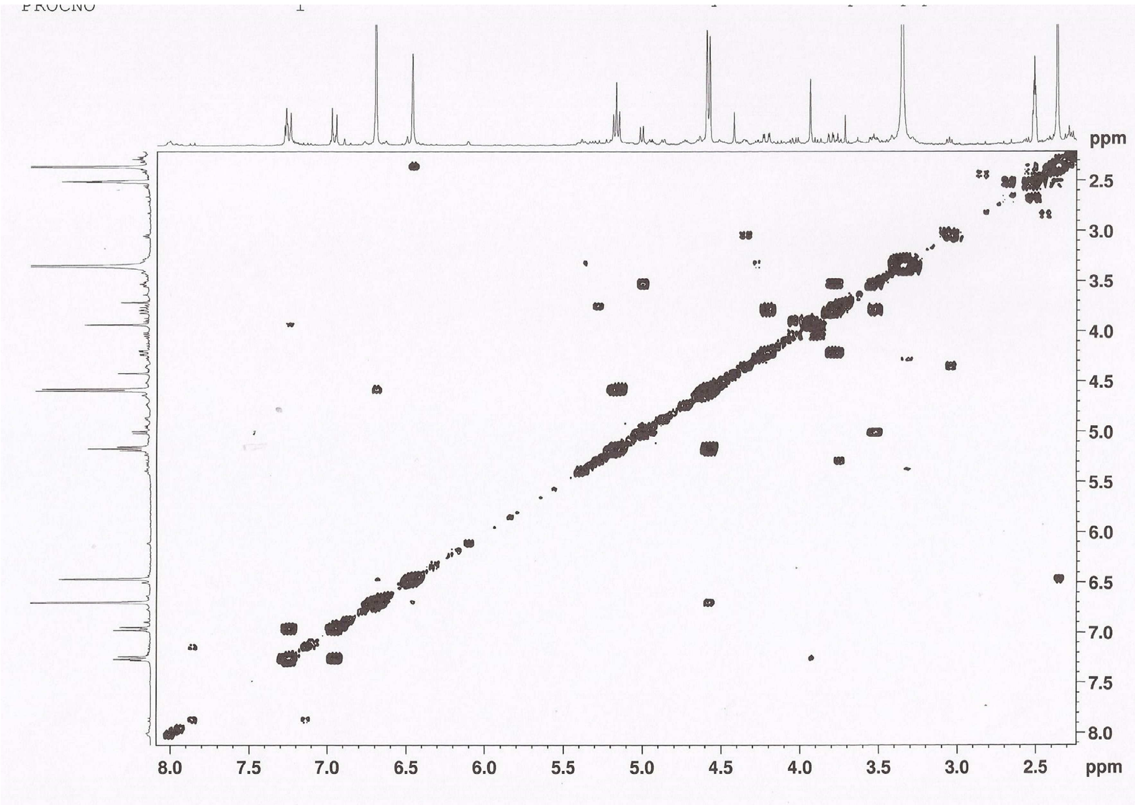

Figure S16. HSQC spectrum of **2** (DMSO-d$_6$, 300.13 MHz) before purification.

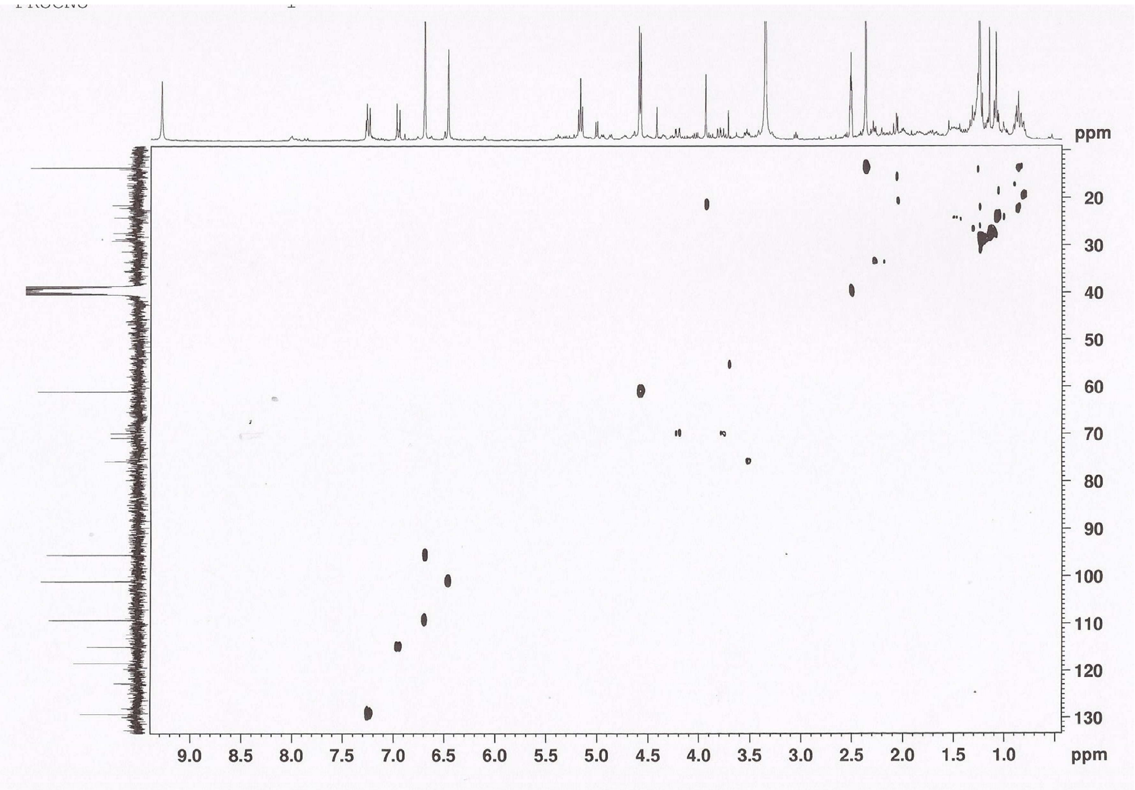

Figure S17. HMBC spectrum of **2** (DMSO-d$_6$, 300.13 MHz) before purification.

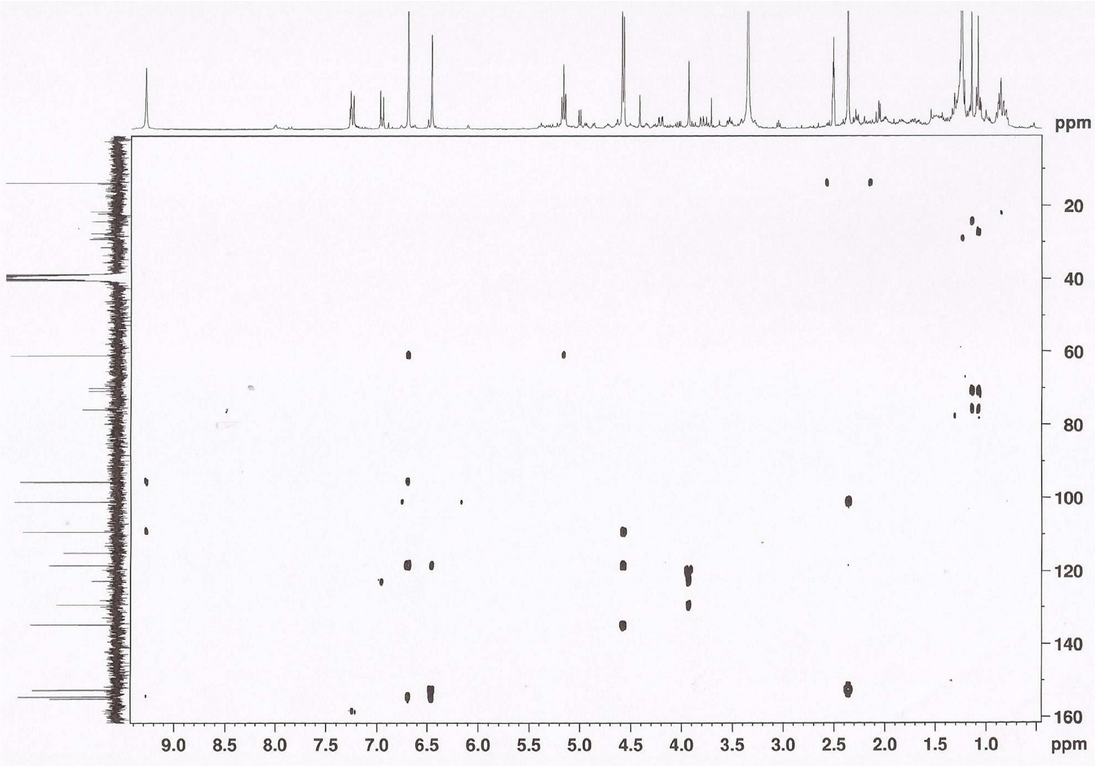

Figure S18. ^{1}H NMR spectrum of **2** (DMSO-d$_6$, 300.13 MHz) after purification.

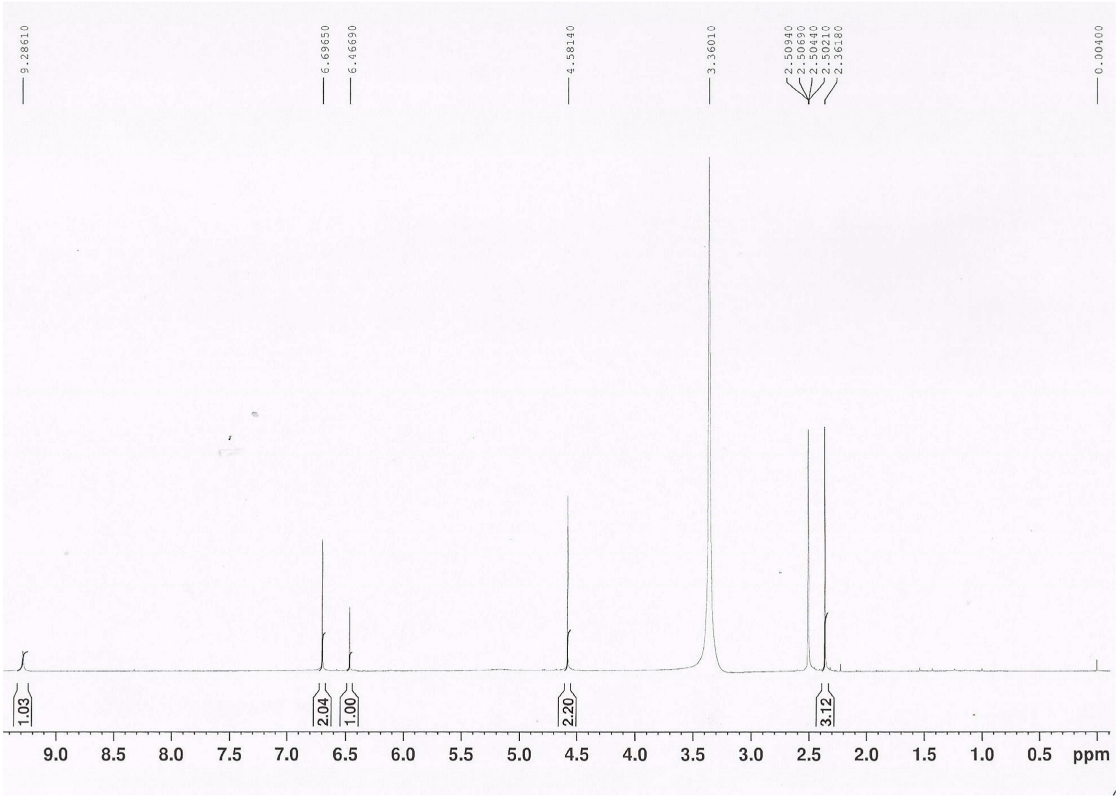

Figure S19. ^{13}C NMR spectrum of **2** (DMSO-d$_6$, 75.4 MHz) after purification.

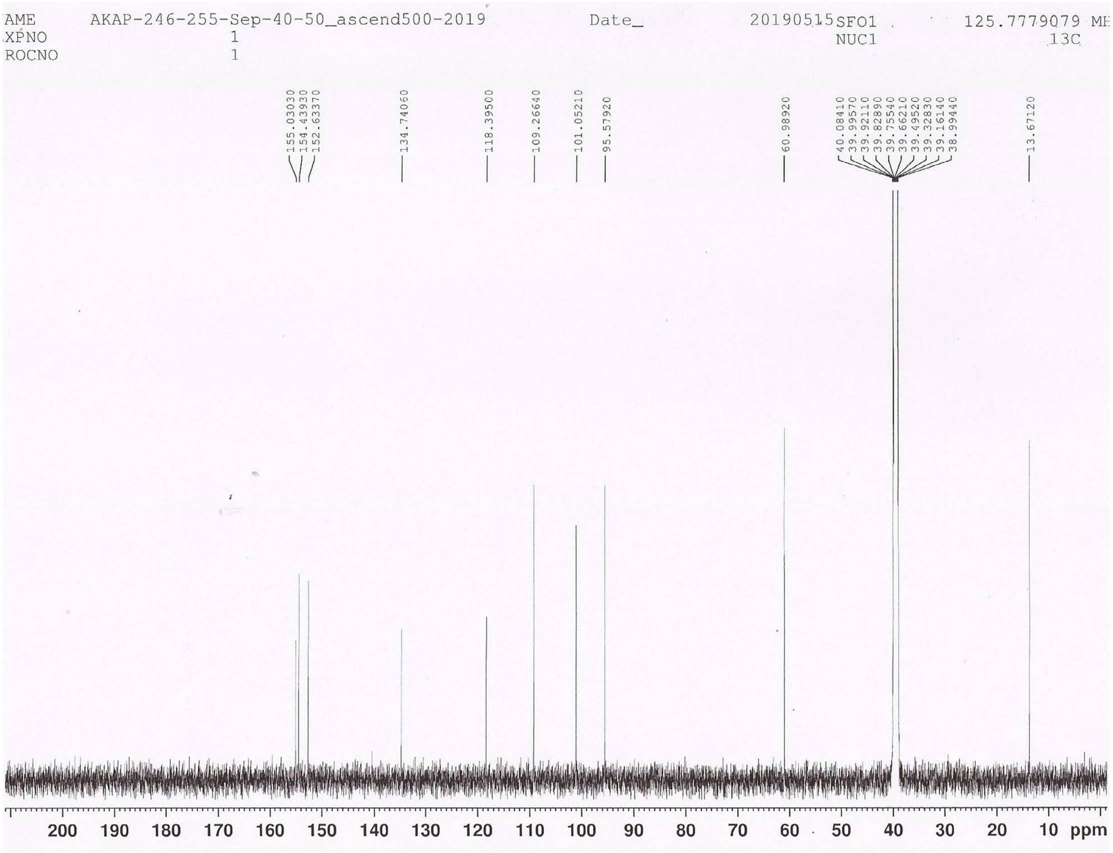

Figure S20. HSQC spectrum of **2** (DMSO-d$_6$, 300.13 MHz) after purification.

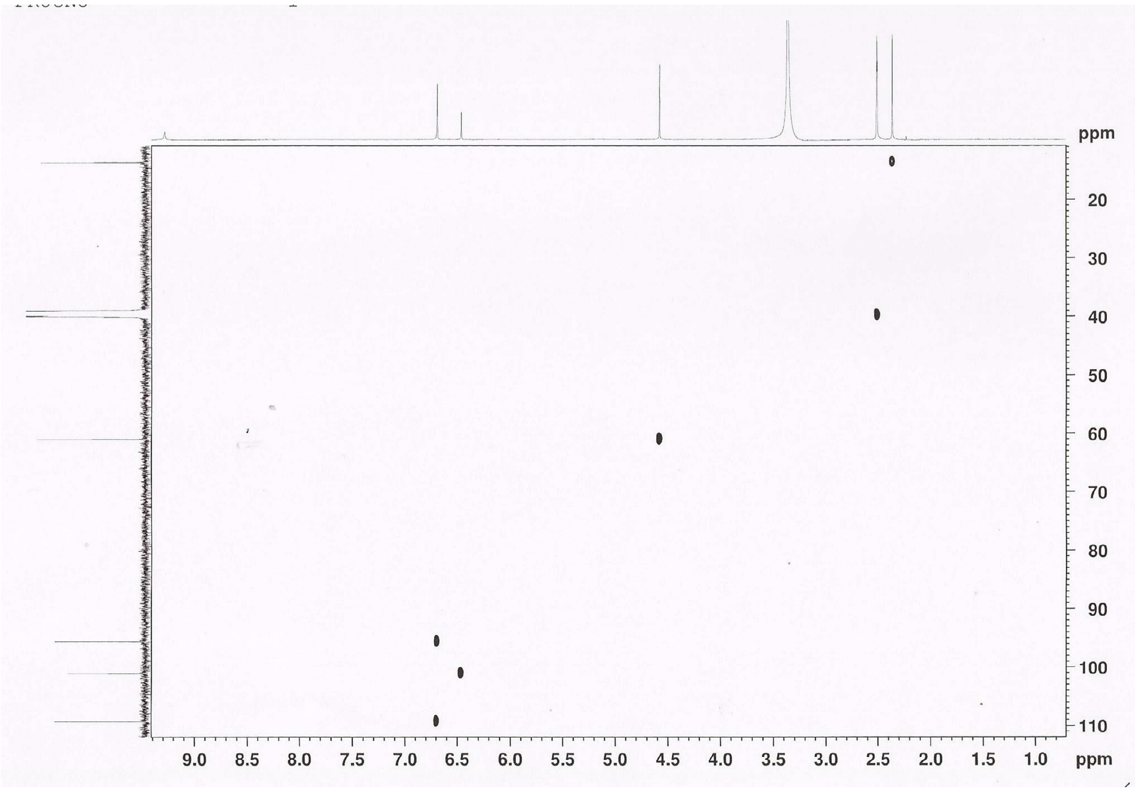

Figure S21. HMBC spectrum of **2** (DMSO-d$_6$, 300.13 MHz) after purification.

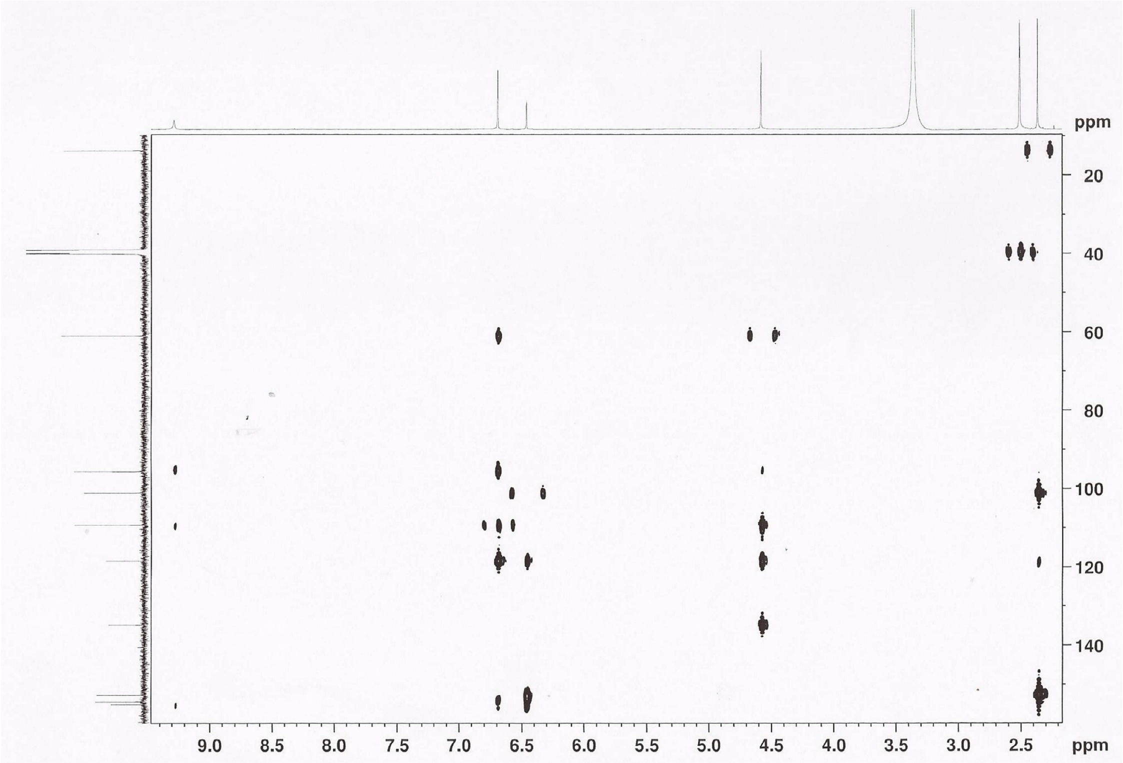

Figure S 22. (-)-HRESIMS of **2** after purification.

Negative Ion Mode

Elemental Composition Report [M-H]$^-$

Single Mass Analysis
Tolerance = 50.0 PPM / DBE: min = -1.5, max = 100.0
Element prediction: Off
Number of isotope peaks used for i-FIT = 3

Monoisotopic Mass, Even Electron Ions
12 formula(e) evaluated with 1 results within limits (up to 20 closest results for each mass)
Elements Used:
C: 10-10 H: 0-150 O: 0-30

Minimum:				-1.5				
Maximum:		10.0	50.0	100.0				
Mass	Calc. Mass	mDa	PPM	DBE	i-FIT	Norm	Conf(%)	Formula
177.0556	177.0552	0.4	2.3	6.5	513.1	n/a	n/a	C10 H9 O3

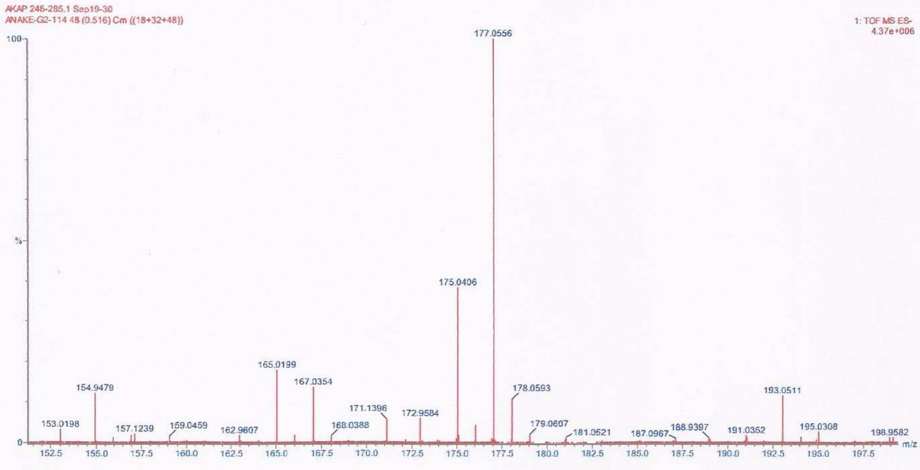

Figure S23. ^{1}H NMR spectrum of **3** (DMSO-d$_6$, 500 MHz).

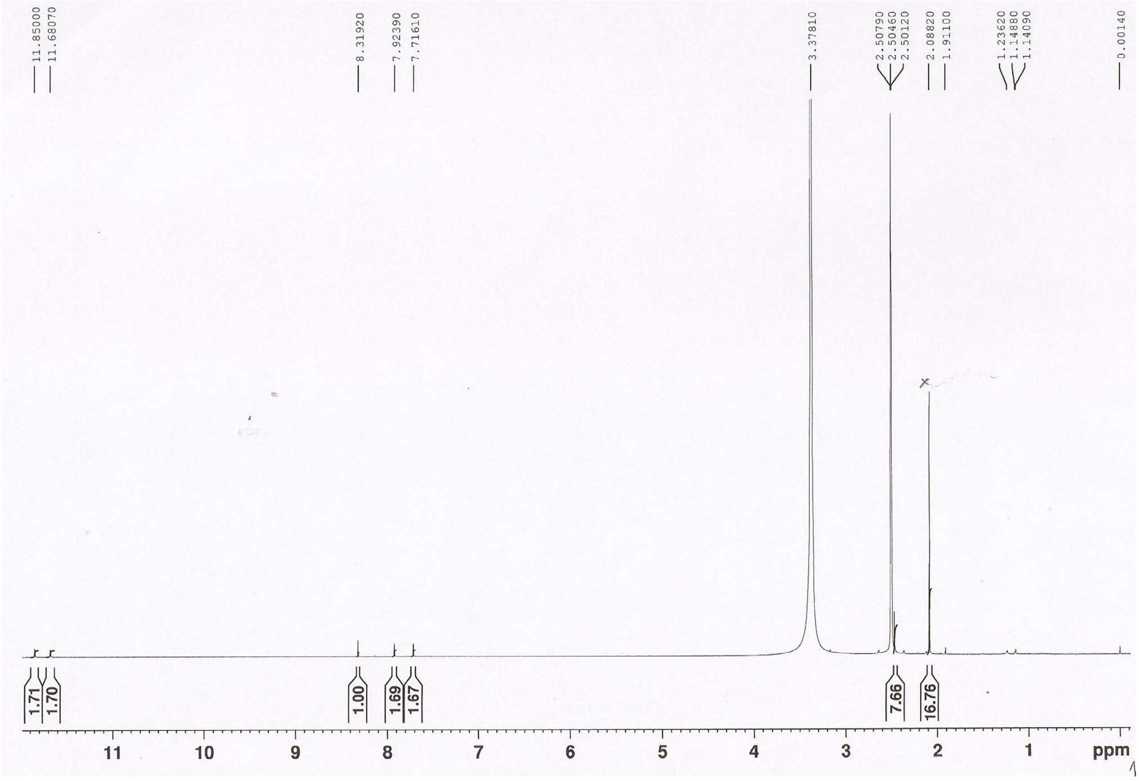

Figure S24. ^{13}C NMR spectrum of **3** (DMSO-d$_6$, 125 MHz).

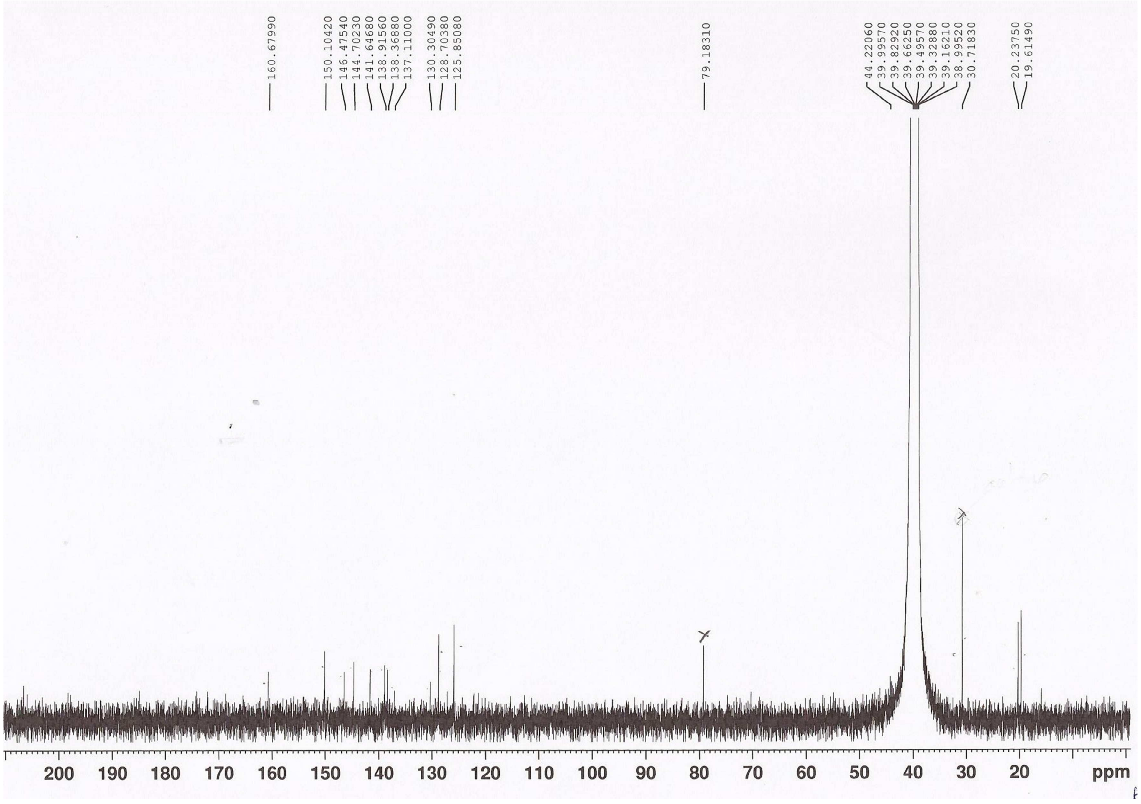

Figure S25. DEPT 135ºspectrum of **3** (DMSO-d_6, 125 MHz).

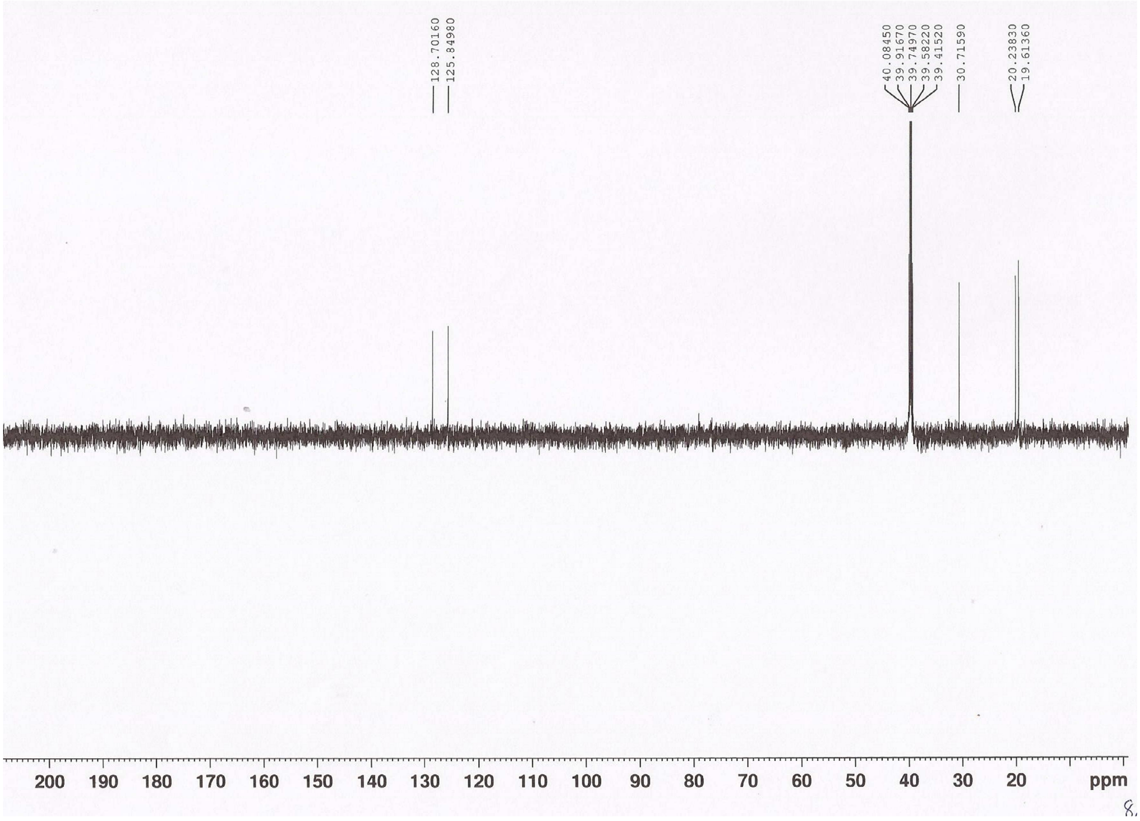

Figure S26. DEPT 90ºspectrum of **3** (DMSO-d_6, 125 MHz).

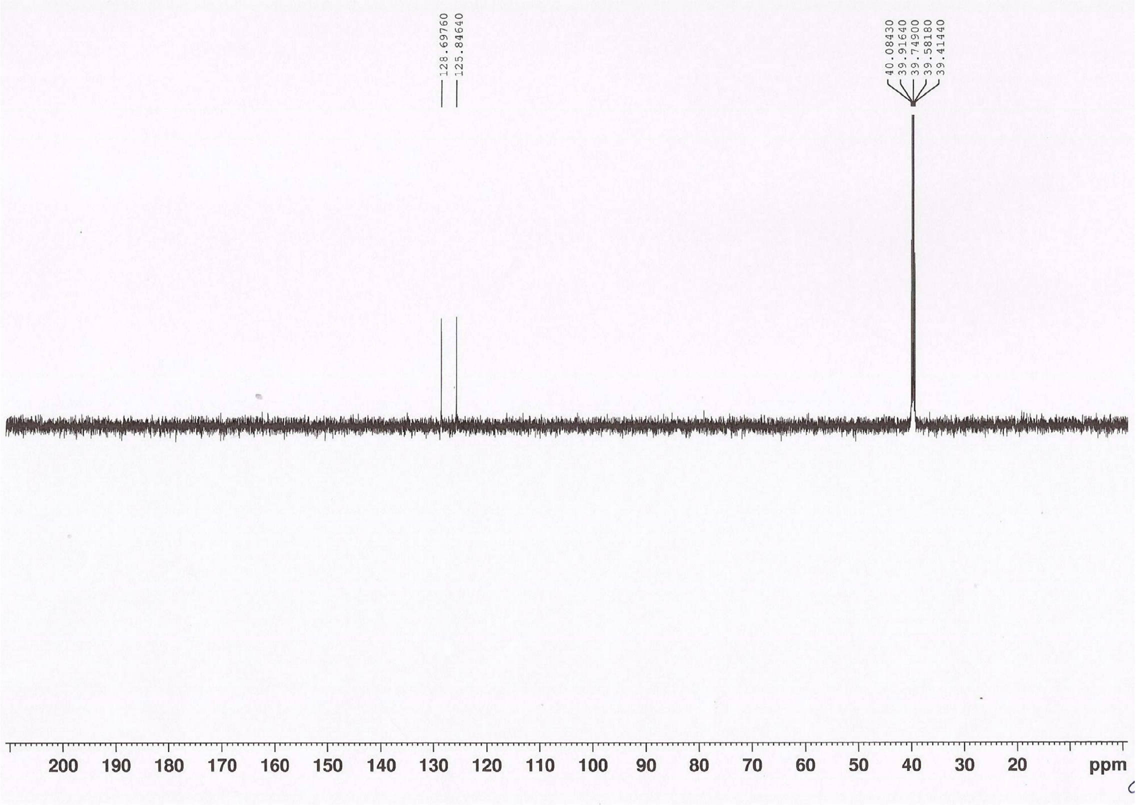

Figure S27. HSQC spectrum of **3** (DMSO-d6, 125 MHz).

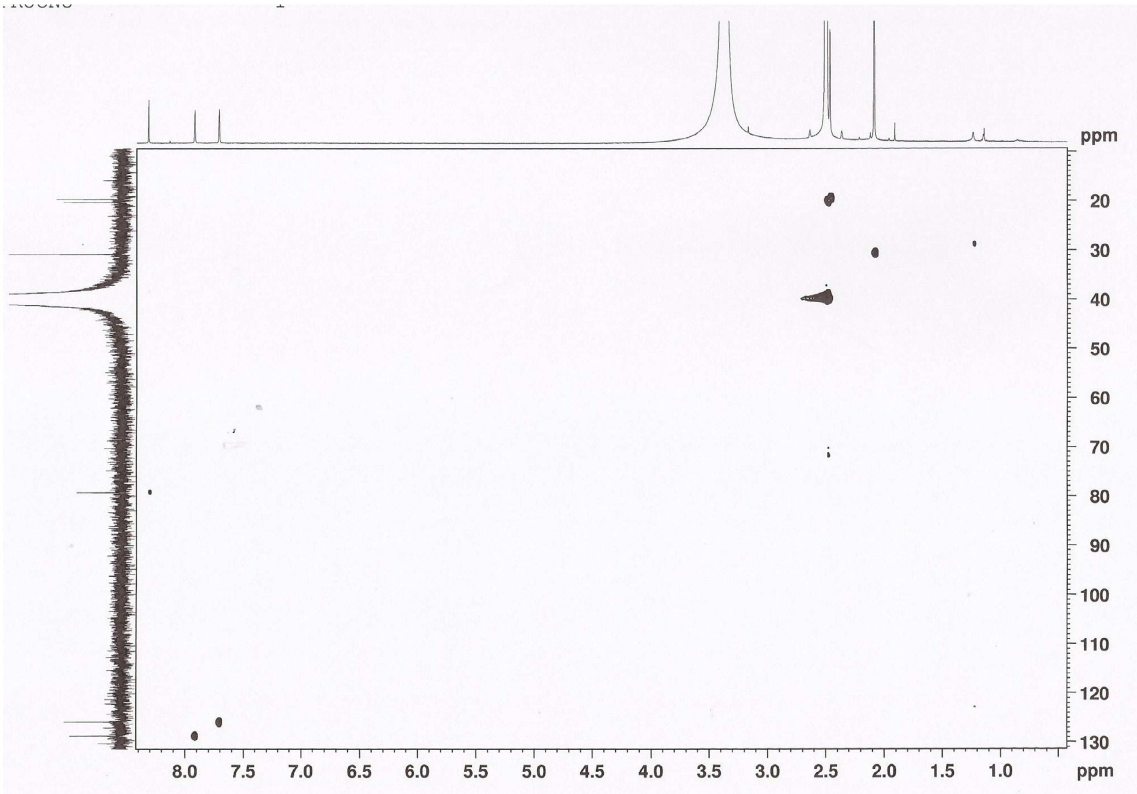

Figure S28. HMBC spectrum of **3** (DMSO-d6, 125 MHz).

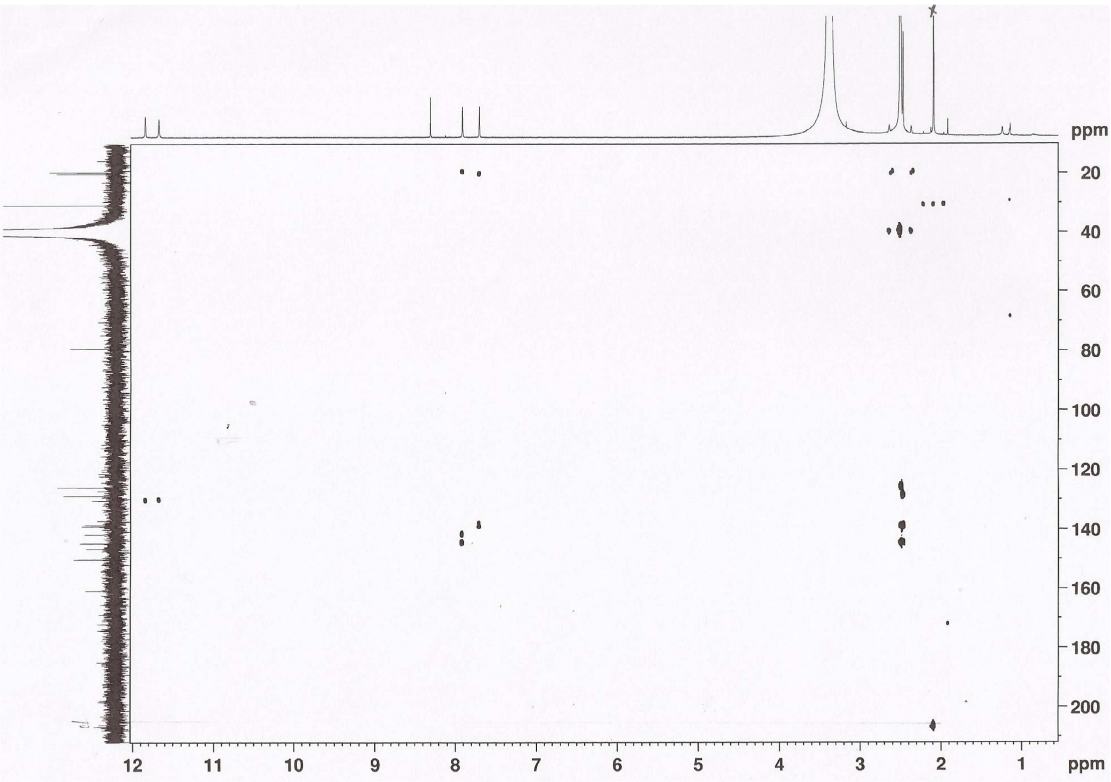

Figure S 29. (+)-HRESIMS of **3**.

Elemental Composition Report [MH]⁺

Single Mass Analysis
Tolerance = 5.0 PPM / DBE: min = -1.5, max = 100.0
Element prediction: Off
Number of isotope peaks used for i-FIT = 3

Monoisotopic Mass, Even Electron Ions
147 formula(e) evaluated with 1 results within limits (up to 20 closest results for each mass)
Elements Used:
C: 12-12 H: 0-150 N: 0-30 O: 0-30

Minimum:				-1.5				
Maximum:		10.0	5.0	100.0				
Mass	Calc. Mass	mDa	PPM	DBE	i-FIT	Norm	Conf(%)	Formula
243.0888	243.0882	0.6	2.5	9.5	778.2	n/a	n/a	C12 H11 N4 O2

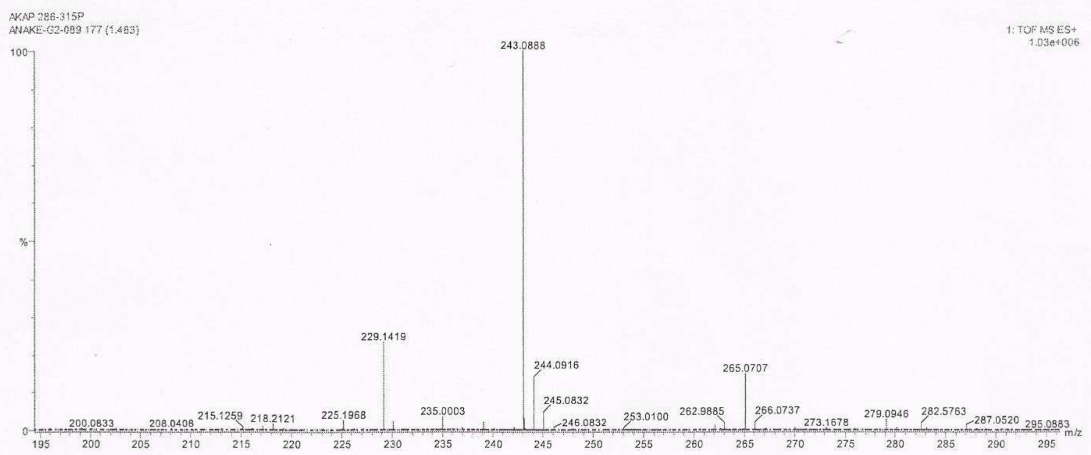

Figure S 30. ^{1}H NMR spectru6 of **4** ($CDCl_3$, 300.13 MHz).

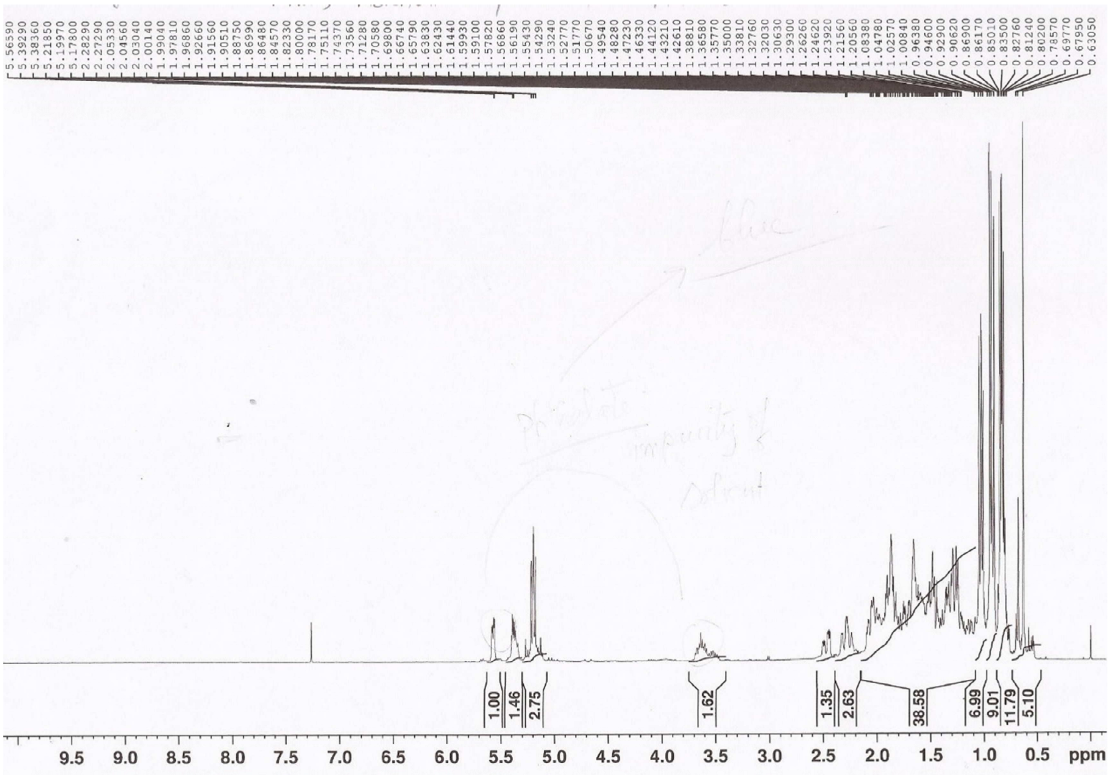

Figure S31. ^{13}C NMR spectrum of **4** ($CDCl_3$, 75.4 MHz).

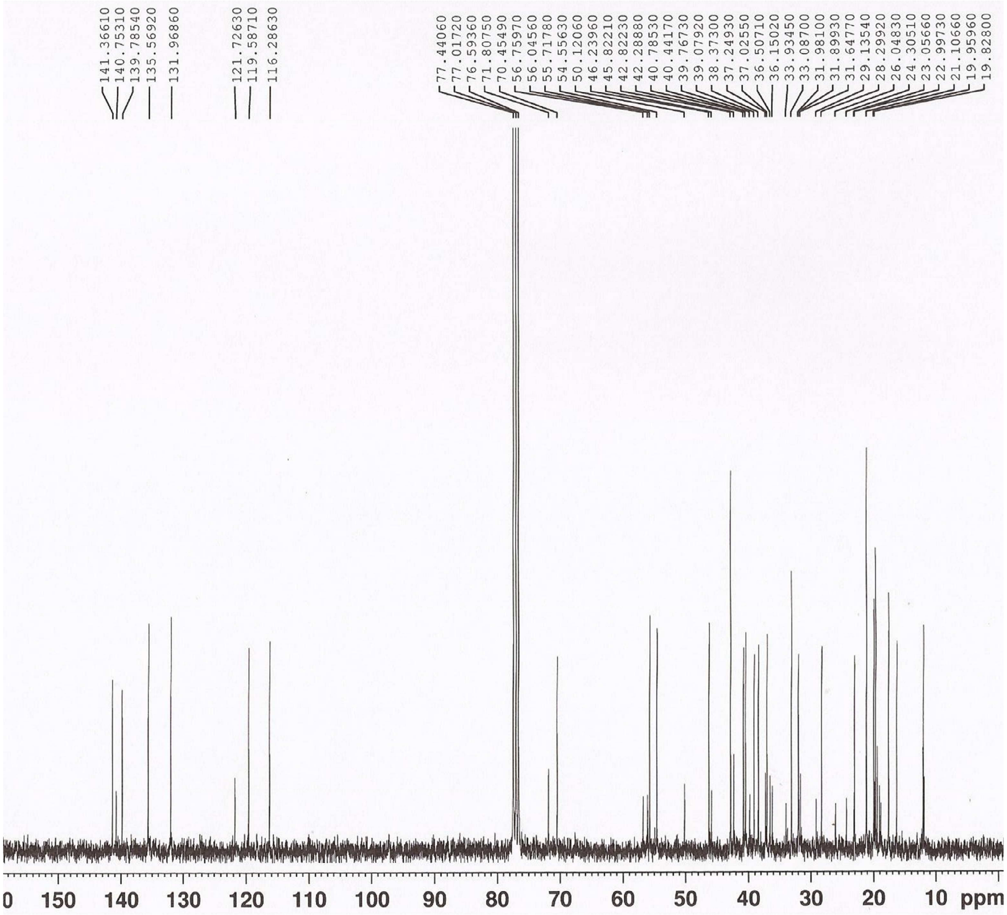

Figure S32. ^{1}H NMR spectrum of **5** ($CDCl_3$, 300.13 MHz).

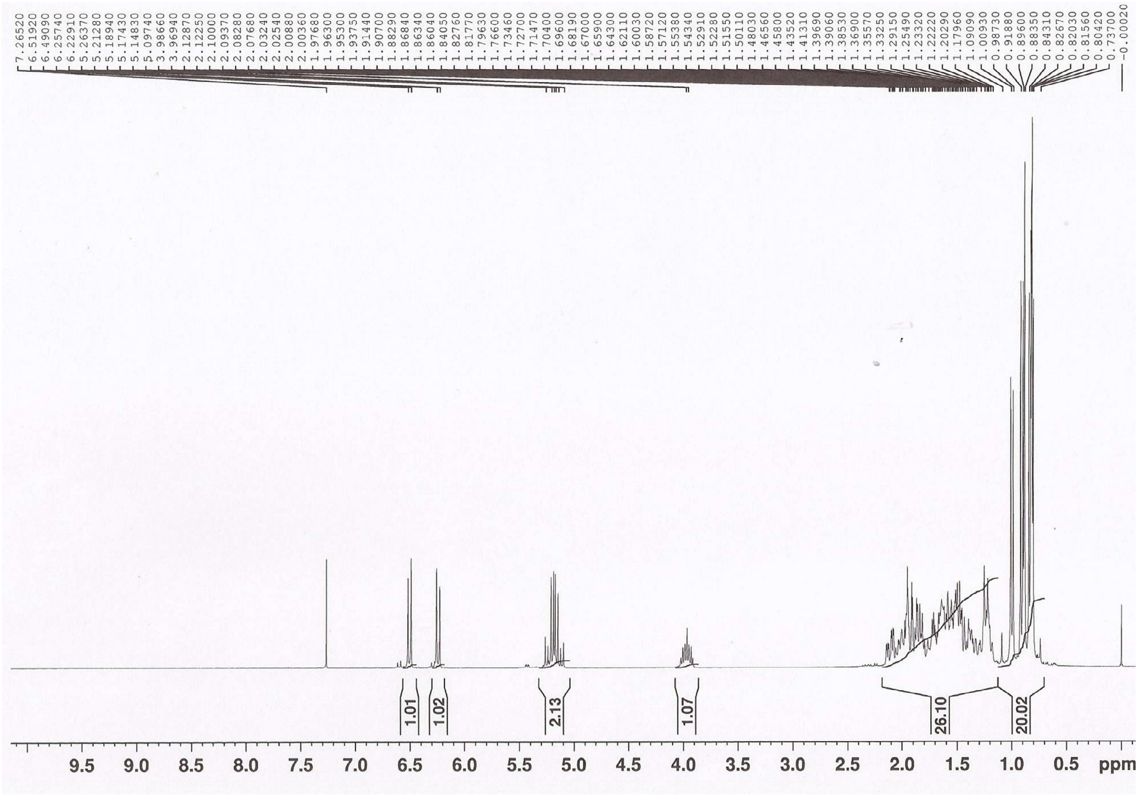

Figure S33. ^{1}H NMR spectrum of **5** ($CDCl_3$, 300.13 MHz).

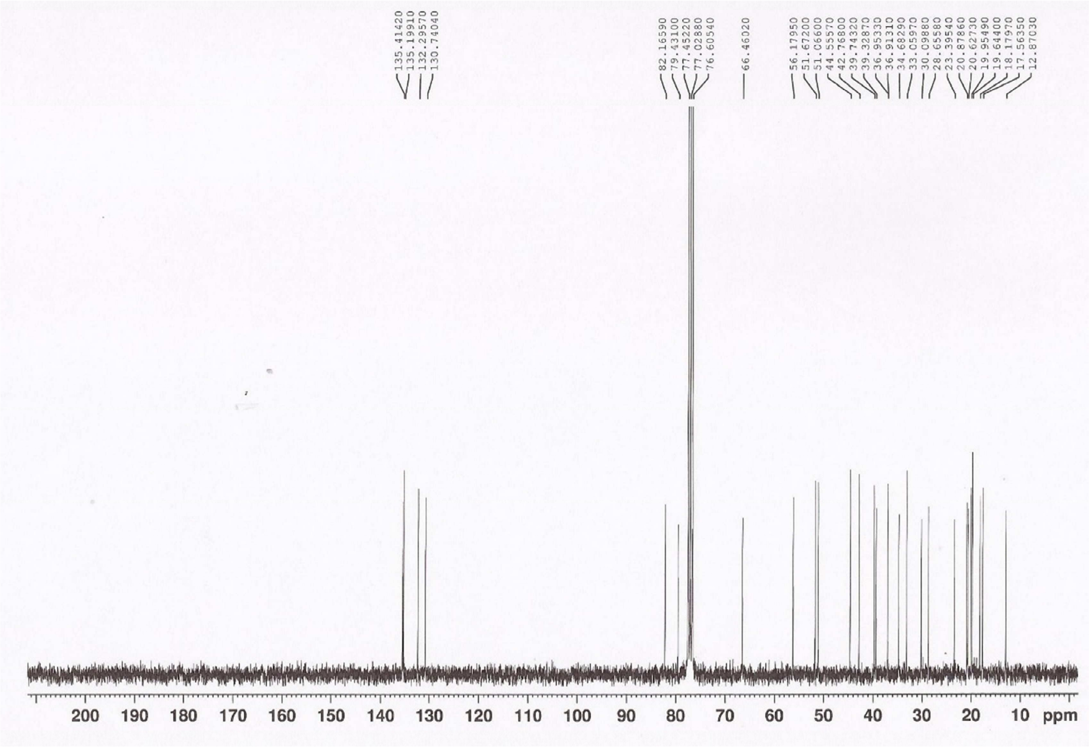

Table 1S. ^{1}H and ^{13}C NMR (DMSO-d_6, 500 and 125 MHz) for lumichrome (**3**).

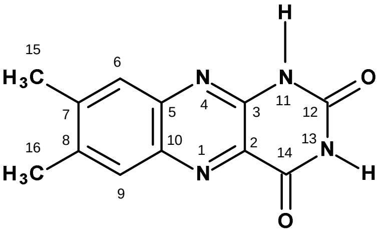

Position	δ_C, type	δ_H, (*J* in Hz)
2	130.2, C	-
3	146.5, C	-
5	138.9, C	-
6	128.7, CH	7.92, s
7	138.3, C	-
8	144.7, C	-
9	125.8, CH	7.71, s
10	141.6, C	-
12	150.1, CO	-
14	160.7, CO	-
15	19.6, CH_3	2.47, s
16	20.2, CH_3	2.50, s
NH-11	-	11.84, brs*
NH-13	-	11.69, brs*

*interchangeable
